# 1-Mesityl-3-(3-Sulfonatopropyl) Imidazolium Protects Against Oxidative Stress and Delays Proteotoxicity in *C. elegans*

**DOI:** 10.3389/fphar.2022.908696

**Published:** 2022-05-24

**Authors:** Natalia Andersen, Tania Veuthey, María Gabriela Blanco, Gustavo Fabian Silbestri, Diego Rayes, María José De Rosa

**Affiliations:** ^1^ Instituto de Investigaciones Bioquímicas de Bahía Blanca (INIBIBB) CCT UNS-CONICET, Bahía Blanca, Argentina; ^2^ Departamento de Biología, Bioquímica y Farmacia, Universidad Nacional Del Sur (UNS), Bahía Blanca, Argentina; ^3^ Departamento de Química, INQUISUR, Universidad Nacional Del Sur, UNS-CONICET, Bahía Blanca, Argentina

**Keywords:** *Caenorhabditis elegans*, imidazolium salts, neurodegenerative disease, proteotoxicity, oxidative stress

## Abstract

Due to the increase in life expectancy worldwide, age-related disorders such as neurodegenerative diseases (NDs) have become more prevalent. Conventional treatments comprise drugs that only attenuate some of the symptoms, but fail to arrest or delay neuronal proteotoxicity that characterizes these diseases. Due to their diverse biological activities, imidazole rings are intensively explored as powerful scaffolds for the development of new bioactive molecules. By using *C. elegans,* our work aims to explore novel biological roles for these compounds. To this end, we have tested the *in vivo* anti-proteotoxic effects of imidazolium salts*.* Since NDs have been largely linked to impaired antioxidant defense mechanisms, we focused on 1-Mesityl-3-(3-sulfonatopropyl) imidazolium (MSI), one of the imidazolium salts that we identified as capable of improving iron-induced oxidative stress resistance in wild-type animals. By combining mutant and gene expression analysis we have determined that this protective effect depends on the activation of the Heat Shock Transcription Factor (HSF-1), whereas it is independent of other canonical cytoprotective molecules such as abnormal Dauer Formation-16 (DAF-16/FOXO) and Skinhead-1 (SKN-1/Nrf2). To delve deeper into the biological roles of MSI, we analyzed the impact of this compound on previously established *C. elegans* models of protein aggregation. We found that MSI ameliorates β-amyloid-induced paralysis in worms expressing the pathological protein involved in Alzheimer’s Disease. Moreover, this compound also delays age-related locomotion decline in other proteotoxic *C. elegans* models, suggesting a broad protective effect. Taken together, our results point to MSI as a promising anti-proteotoxic compound and provide proof of concept of the potential of imidazole derivatives in the development of novel therapies to retard age-related proteotoxic diseases.

## 1 Introduction

During the last century, scientific advances have accomplished an increase in the life expectancy of the population. This achievement, however, entails new challenges such as a higher prevalence of age-related disorders. Today, neurodegenerative diseases (NDs) such as Alzheimer’s disease (AD), Parkinson’s disease (PD) and Huntington’s disease (HD), have become a major health concern. Despite decades of intensive investigation, these diseases still lack a cure. While each of these disorders has its distinctive features (e.g., the type of neurons affected), they share a common pathological hallmark: the abnormal formation and accumulation of misfolded protein aggregates in specific neurons (Gitler et al., 2017). In AD, the senile plaques mainly consist of regular β-sheet fibrils of β-amyloid peptide (Aβ); in PD, the Lewis bodies are composed of the protein α-synuclein (α-syn); in HD, an abnormally expanded polyglutamine domain of the protein huntingtin induces protein aggregation (Morfini et al., 2005; Huang and Mucke, 2012). Due to the presence of these typical protein aggregates that cause neuronal damage, these diseases are called proteinopathies.

The cellular and molecular mechanisms underlying protein aggregation are not yet understood (Bucciantini et al., 2002; Sweeney et al., 2017). However, protective proteins involved in modulating scavenging mechanisms for free radicals and oxidative metabolites, such as the cytoprotective transcription factors (TFs) Nrf-2 (Nuclear factor erythroid 2-related factor 2), HSF (Heat Shock Transcription Factor) and FOXO (Forkhead box) were found to be actively involved in neuronal proteostasis (Hasanbašić et al., 2018; Nandi et al., 2019). There is strong evidence that saturation of these endogenous antioxidant defenses represents key factors in the progression of NDs (Jenner, 2003; Blesa et al., 2015; Lévy et al., 2019). For instance, it was reported that overexpression of active forms of HSF-1 decreases protein aggregation both *in vitro* and *in vivo* models of NDs (Fujimoto et al., 2005; Kumsta et al., 2017). In addition, while downregulation of Nrf-2 activity has been shown to induce α-syn aggregation (He et al., 2013), its expression is neuroprotective in cellular and animal models of PD (Lastres-Becker et al., 2012; Fu et al., 2018). Moreover, FOXO activity delays the onset of protein aggregation in animal models of HD and AD (Jiang et al., 2011; Hesp et al., 2015). Hence, modulating the pathways controlled by these TFs may inhibit or at least retard pathological protein aggregation.

Since the 1970s, the free-living nematode *C. elegans* has been extensively used as an inexpensive, safe and powerful organism for basic and applied biomedical studies. In the last 20 years, several reports also highlighted the use of this nematode for modeling human pathologies (including NDs) and for the screening of potential therapeutic compounds (Kaletta and Hengartner, 2006; Markaki and Tavernarakis, 2010; Giunti et al., 2021). Its short and genetically tractable lifespan makes *C. elegans* particularly suitable for exploring age-related diseases (Kenyon et al., 1993; Samuelson et al., 2007; Uno and Nishida, 2016). In addition, the analysis of synaptic connections by serial electron microscopy has led to the reconstruction of the complete neural wiring diagram (White et al., 1986; Cook et al., 2019), which allows the identification of precise neural circuits that control specific behaviors and physiological processes. Moreover, *C. elegans* transparency permits the visualization of the morphology and activity of specific cells using transgenic reporters and genetically encoded calcium indicators (Chalfie et al., 1994; Kerr and Schafer, 2006; Chung et al., 2013) in freely moving animals. Given the striking conservation in neuronal function throughout the animal kingdom, *C. elegans* offers the possibility to provide fundamental insights into nervous system (NS) aging. It has been shown that the NS of *C. elegans* undergoes age-dependent decline with morphological structural alterations comparable to those that take place in the human brain (Burke and Barnes, 2006; Benard and Hobert, 2009; Fjell and Walhovd, 2010; Bénard and Doitsidou, 2016; Hess et al., 2019).

*C. elegans* has no orthologous gene for Aβ, α-syn, or huntingtin. However, transgenic animals that express the disease-causing protein for AD, PD, and, HD either in body-wall muscle cells, in all neurons, or in a specific subset of neurons have been generated (Link, 1995; Morley et al., 2002; Brignull et al., 2006; Witan et al., 2008; Gidalevitz et al., 2009; Liachko et al., 2010). In most cases, the protein is tagged with a fluorescent reporter, which permits monitoring protein aggregation in whole live animals. In addition, genetic approaches in *C. elegans* have pioneered the elucidation of highly conserved cellular pathways controlling proteostasis, oxidative stress (OS) resistance, and lifespan (Fontana et al., 2010; Kenyon, 2010). The best-known example is the DAF-2/insulin/insulin-like growth factor signaling (IIS) pathway, which regulates lifespan and proteotoxic stress resistance from worms to flies to mammals (Cohen and Dillin, 2008; Murphy and Hu, 2013; Altintas et al., 2016). The DAF-2/IIS pathway is a crucial regulator of conserved cytoprotective mechanisms such as DAF-16/FOXO, SKN-1/ Nrf-2, and the HSF-1/HSF1. Similar to mammals, activation of these mechanisms reduces the damage induced by protein aggregation in *C. elegans* models of NDs (Kenyon et al., 1993; Hsu et al., 2003; Cohen et al., 2006; Anckar and Sistonen, 2011; Teixeira-Castro et al., 2011; Zhang et al., 2012; Steinbaugh et al., 2015). Thus, *C. elegans* has evolved as an excellent whole-animal platform to identify genes, drug targets, and chemical compounds with plausible neuroprotective roles for human NDs (Morley et al., 2002; Lakso et al., 2003; Kang and Avery, 2009; Yerbury et al., 2016; Sanchis et al., 2019; Giunti et al., 2021; Pereira et al., 2022).

To develop new bioactive molecules, the pharmaceutical industry has extensively benefited from the versatility of heterocyclic compounds (Gaba et al., 2014). Due to their capacity to bind to several biological targets, nitrogen-containing heterocycles such as imidazole rings, have been largely used as powerful scaffolds for drug development (Sharma et al., 2016). A wide spectrum of activities, including analgesic, anti-inflammatory, anti-infection, anticonvulsant, and antiparasitic actions have been described for imidazole-containing compounds (Blanco et al., 2018; Shalmali et al., 2018). In addition, the imidazole ring’s biocompatibility together with the feasibility to synthesize new derivatives with different pharmacokinetics and pharmacodynamics properties by chemical modifications further enhance their potential use in drug development (Anderson and Long, 2010).

In this work, we used *C. elegans* to evaluate the anti-proteotoxic potential of imidazole derivatives. We found that exposure to one of these derivatives, 1-Mesityl-3 (3-sulfonatopropyl) imidazolium (MSI), induces the HSF-1 pathway leading to an increase in OS resistance. Furthermore, this compound delays the onset of proteotoxic-associated phenotypes in transgenic animals expressing ND-related pathological proteins. In addition, we provide a proof-of-concept validation for the screening of imidazole compounds with anti-proteotoxic activity using *C. elegans* models of protein aggregation.

## 2 Methods

### 2.1 Synthesis: General Procedures

**SCHEME 1:**
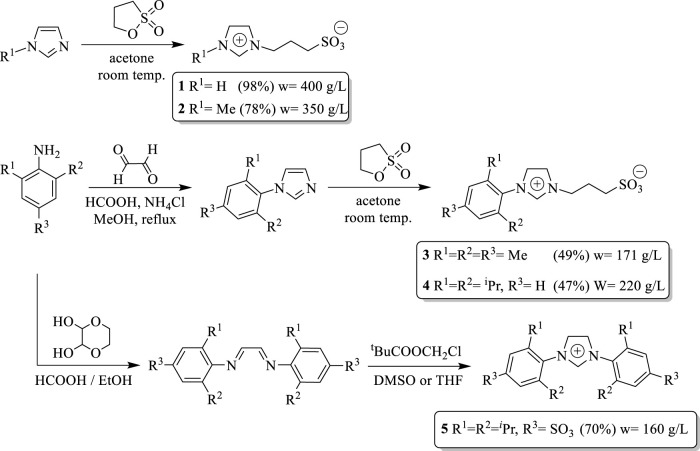
Synthesis of sulfonated-imidazolium salts.

All reactions were carried out under a dry nitrogen atmosphere by using Schlenk techniques. Organic solvents were dried and distilled under nitrogen and degassed before use. All imidazolium salts were synthesized according to reported procedures (Blanco et al., 2018) as shown in Scheme 1 (Spectroscopy data, see Supplementary Material electronic). 1H- and 13C-NMR spectra were recorded on a Bruker ARX 300 (300.1 MHz for 1H, 75.5 MHz for 13C) using D_2_O or DMSO-d6 as solvents. C, H, and N analyses were performed by The Analytical Services of the Universidad Nacional del Sur (Argentina) with an Exeter Analytical Inc. CE-440 microanalyzer.

### 2.2 Determination of Oxidation-Reduction Potential

The Oxidation-Reduction Potential (ORP) of the complexes was calculated using an Ion-selective pH meter Thermo Scientific Orion 720 A. Electrode Redox/ORP Thermo Scientific Orion 9678BNWP.

### 2.3 *C*. *elegans* Culture and Maintenance

Worms were cultured and maintained on Nematode Growth Medium (NGM) agar plates seeded with *Escherichia coli* OP50 as a food source (Brenner, 1974; Stiernagle, 2006). Worm culture and all the experiments were carried out at 20°C, unless otherwise noted. The wild-type strain was Bristol N2. The strains were provided by the *Caenorhabditis* Genetics Center (CGC), which is funded by the NIH Office of Research Infrastructure Programs (P40 OD010440).

Low population density was maintained throughout development and during the assays. For all experiments, animals were age-synchronized. The strains used were: CL2070 *dvIs70[Phsp-16.2::gfp + pRF4]*, CL2166 *dvIs19[Pgst−4::gfp::NLS]*, TJ356 *zIs356[Pdaf-16::DAF−16a/b::gfp + pRF4]*, NL5901 *pkIs2386[Punc−54::alphasynuclein::yfp + unc−119*(*+*)], CL2006 *dvIs2[pCL12*(*unc−54/human Aβ peptide 3–42 minigene*) *+ rol−6(su1006)*], AM141 *rmIs133[Punc−54::Q40::yfp]*, PS3551 *hsf-1* *(sy441)* I, QV225 *skn-1* *(zj15)* IV, GR1307 *daf−16(mgDf50)* I.

### 2.4 Imidazolium Salts Exposure

The pharmacological assays were performed as described before (Blanco et al., 2018; De Rosa et al., 2019). 10 mM individual imidazole derivatives stocks were prepared with sterile MilliQ water and diluted into NGM agar at 10, 50 or 100 μM. The control condition was prepared with sterile MilliQ without the compound. Plates were stored at 4°C and used within 1 week.

In all experiments, age-synchronized individuals were used. To achieve this synchronization, gravid adults were placed on NGM plates containing the drug, allowed to lay eggs for 8 h, and then removed. Descendants were maintained on these plates until the stage of interest.

### 2.5 Oxidative Stress Resistance Assay

Iron sulfate (FeSO_4_) was used as an oxidative stressor as previously reported (De Rosa et al., 2019). Briefly, 20 L4 worms were transferred to 35-mm agar plates (3 plates per condition) containing 10 mM FeSO_4_. After 60 min, animal survival was immediately scored. Worms were scored as dead if they failed to respond to prodding with a platinum wire pick to the nose.

### 2.6 Pharyngeal Pumping Rate

Age synchronized animals were grown on NGM plates containing 10 μM of the compound. Young adults (24 h post L4s) were transferred to new NGM plates containing the drug and seeded with fresh bacteria. The number of contractions in the terminal bulb of the pharynx was video-recorded for 15 s using a stereomicroscope coupled to NIKON DS-Qi2 Camera (100x magnification). Recordings were reproduced at a slow speed to manually count the number of pharyngeal pumps. This experiment was independently repeated three times.

### 2.7 Developmental Rate Evaluation

A short synchronization was performed. Briefly, 4 gravid adults were placed on NGM plates (4 plates per condition), left to lay eggs for 3 h and removed. The number of animals at different developmental stages (L1-L3, L4, and adults) was counted at 24, 48, and 72 h. Results were expressed as the number of animals at a given stage × 100/ Total number of living worms at that time point. This experiment was independently repeated three times.

### 2.8 Microscopy and Image Analysis

For microscopy analysis, animals were grown on NGM plates containing the compound. At the proper age for each experiment, worms were immobilized in 25 mM Levamisole (in M9) and mounted onto slides with 2% agarose pads. Images were obtained using an epifluorescence microscope (NIKON ECLIPSE TE2000-S) coupled to a monochrome CMOS Nikon DS-Qi2 Camera (NIS-Elements acquisition software) at × 10 and × 20 objective mag. Images were analyzed using Image J FIJI software (ImageJ, National Institutes of Health, Bethesda, MD, United States).

### 2.9 *hsp-16.2* Expression

*hsp-16.2* expression was analyzed in a transgenic strain containing a transcriptional GFP reporter in a wild-type background CL2070 *dvIs70[Phsp−16.2::gfp + pRF4]*.

Briefly, 16 h post-L4 animals (adult day 1) were subjected to heat shock for 2 h at 35°C followed by 4 h of recovery at 20°C. Upon induction by thermal stress, GFP fluorescence was detectable at the head of the worm, including the pharynx (Strayer et al., 2004; Ogawa et al., 2016). Therefore, images were taken in this region, and *hsp-16.2*::GFP fluorescence intensity was measured in same-sized regions of the pharynx, specifically at the terminal bulb using ImageJ FIJI software. Results were normalized to control conditions (without MSI).

### 2.10 Worm Paralysis Assay

Paralysis was evaluated in transgenic animals expressing the human amyloid β_3-42_ (Aβ3_−42_) in body wall muscle cells (construct [*Punc-54::human A-beta 3–42; pRF4* (*rol-6(su1006)*], see CL2006 in strain list). This strain experiments age-dependent paralysis (Link, 1995), which was assessed under basal conditions (paralysis assay) and after a brief heat-shock of 35°C for 30 min (thermal-induced paralysis assay) (Srivastava et al., 2008; Arya et al., 2009; Regitz et al., 2016).

#### 2.10.1 Paralysis Assay

Animals were synchronized by placing gravid animals in NGM plates, and after 12 h of egg-laying, the adults were removed. After 3.5 days post-hatch, animals were re-synchronized by picking L4 animals. From that moment onwards, worms were tested every day for paralysis by prodding with a platinum wire. Animals were scored as paralyzed if they failed to move their bodies or only moved their head (without rolling). Paralyzed animals were removed from plates for the next day’s reading (Cohen et al., 2006).

#### 2.10.2 Thermal-Induced Paralysis Assay

Heat shock accelerates the paralyzation process (Srivastava et al., 2008; Arya et al., 2009; Regitz et al., 2016). In this case, adult day 1 worms were subject to brief heat stress (35°C for 30 min), and paralyzed animals were subsequently scored as described previously.

### 2.11 Locomotion Assays

To evaluate mobility, one animal (at the indicated days of adulthood, starting at day 0 (L4 stage)) was transferred to a multi-well plate containing 150 μl of M9 buffer (one worm/well). After 1 min of habituation, thrashing rates were manually counted under a dissecting scope as previously described (Jones et al., 2011; Agotegaray et al., 2020). A single thrash is defined as a change in the direction of bending at the midbody (Buckingham and Sattelle, 2009; Jones et al., 2011). During this assay, the number of times that the concavity of the worm is directed to the same side (e.g., concavity to the left) is counted. This number is then multiplied × 2 (to include the number of times that the body concavity faces the right side) to get the number of thrashes per minute. All assays were carried out by two independent operators and were blinded to the sample identities.

### 2.12 Alpha-Synuclein Protein Quantification

Alpha-synuclein aggregation was evaluated at increasing ages using the strain NL5901 *pkIs2386 [Punc−54::α-synuclein::yfp + unc−119*(*+*)]. These animals express alpha-synuclein fused to YFP in body wall muscle cells (van Ham et al., 2008). After their corresponding incubation with MSI, animals were mounted and the YFP signal in the head region was examined under a fluorescence microscope.

To measure the accumulation of alpha-synuclein, the head area was highlighted with a uniform rectangle, and mean fluorescence intensity was quantified using Image J FIJI software (Jadiya et al., 2011; Jadiya et al., 2016; Tsai et al., 2020). Results were normalized to the control group.

### 2.13 Polyglutamine (Poly-Q) Aggregation Quantification

AM141 strain [P*unc−54::Q40::yfp*] was used for the poly-Q aggregation assay (Morley et al., 2002). At the indicated time, at least 30 worms from each condition were immobilized and mounted, as described above. Images were acquired using fluorescence microscopy. The number of aggregates and their size were quantified with the analyze particle function in ImageJ FIJI software.

### 2.14 *In Silico* Evaluation of ADME Profile, Bioavailability, and Drug-Likeness

SMILE structure for each compound was generated by using the structure file generator, available at the free online tool SwissADME web server (http://www.swissadme.ch/) (Daina et al., 2017). Considering six physicochemical properties such as solubility, molecular size, polarity, lipophilicity, saturation, and flexibility, this tool predicts and weights the probability of the oral bioavailability of the drug by building a bioavailability radar.

The Abbott bioavailability score was also calculated to predict the probability of a 10% oral bioavailability or Caco-2 diffusion (Martin, 2005; Daina et al., 2017).

Human intestinal absorption (HIA) of the compounds was also simulated (Daina and Zoete, 2016). The drug-likeness analysis was carried out using the validated rules used as high-throughput screens filters in leading pharmaceutical companies, including Lipinski (Pfizer), Ghose (Amgen), Veber (GSK), Egan (Pharmacia), and Muegge (Bayer). All rules were evaluated to predict whether a drug is likely to have useful pharmacokinetic properties.

### 2.15 Statistics

For most of our experiments, and in accordance with most reports using *C. elegans*, we used a large number of animals per condition in each assay (more than 40–50 animals). This number of animals is large enough to ensure appropriate statistical power in the test used. All the statistical tests were performed after checking normality. We performed the experiments at least 3 or 4 times to ensure reproducibility. All animals were grown in similar conditions and the experiments were performed on different days, with different animal batches. The results presented in each figure are the mean or median of at least three independent assays, as appropriately indicated. The statistical significance was assessed by performing a Student’s t-test, one-way ANOVA followed by a posthoc test, as appropriate. *p < 0.05, **p < 0.01, ***p < 0.001.

## 3 Results

### 3.1 Two Derivatives, 1-(3-sulfonatopropyl) Imidazolium and 1-Mesityl-3-(3-Sulfonatopropyl) Imidazolium Induce Protection Against Oxidative Stress

We have recently synthesized imidazolium-derived compounds to evaluate their biological activity in *C. elegans*. From an initial set of 11 compounds, we identified 2 with anthelmintic potential (Blanco et al., 2018). All other compounds did not alter nematode survival, even at higher concentrations (Blanco et al., 2018). Different biological effects may result from small differences in the molecular structure of heterocyclic compounds, such as imidazole derivatives (Siwach and Verma, 2021). Therefore, it is likely that these compounds have other biological activities. We decided to evaluate their ability to modulate resistance to oxidative stress (OS). Several molecules or processes that increase resistance to oxidative or thermal stress have been shown to decrease proteotoxicity, including that triggered by proteinopathies (Tsunemi et al., 2012; Chakraborty et al., 2013; Kumar and Devaki, 2015).

**FIGURE 1:**
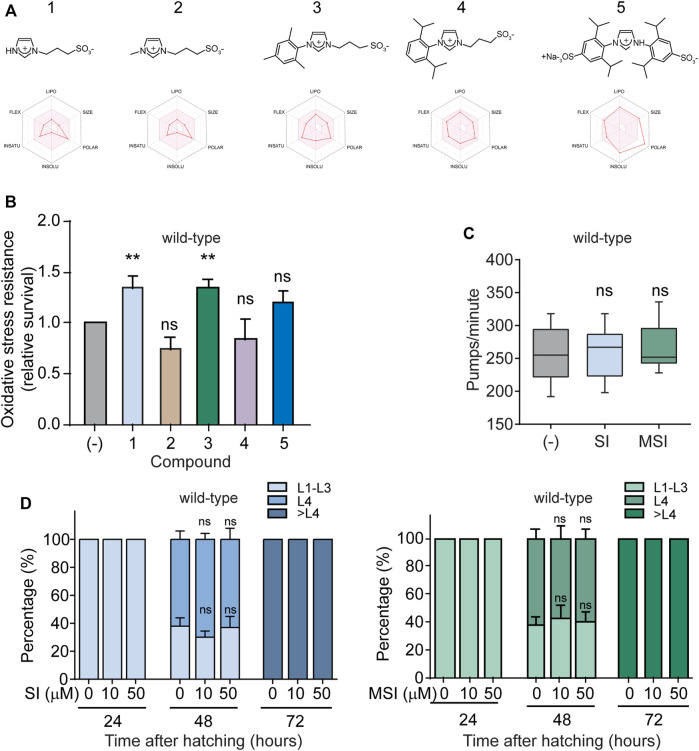
Effect of imidazolium salts on oxidative stress resistance in wild-type *C. elegans*. **(A)**. Chemical structures of imidazolium salts with their corresponding oral bioavailability radar below. The pink area represents the optimal range for each property. Compounds 1: 1-(3-sulfonatopropyl) imidazolium, 2: 1-Methyl-3-(3-sulfonatopropyl) imidazolium, 3: 1-Mesityl-3-(3-sulfonatopropyl) imidazolium, 4: 1-(2,6-Diisopropylphenyl)-3-(3-sulfonatopropyl) imidazolium and 5:1,3-bis(2,6-diisopropyl-4-sodiumsulfonatophenyl) imidazolium. **(B)**. Oxidative stress resistance of wild-type worms in the absence (-) or presence of 10 μM of each imidazolium salt (60–80 animals per condition per experiment, *n* = 3–8). Oxidative stress was induced by 10 mM FeSO4 for 1 h. Survival was scored immediately after this treatment. Data are normalized to the resistance of wild-type animals in the absence of compound assayed the same day. Data in the bar graph are represented as mean ± SEM. Statistical significance was evaluated by One-way ANOVA followed by Holm–Sidak’s test for multiple comparisons against the control group (-) (**p < 0.01). **(C)**. Pharyngeal pumping rates (pumps per min) of wild-type worms in the absence (-) or presence of 10 μM of SI and MSI (30 animals per condition). Data is represented as a box plot with a line at the median. Statistical analysis of the data was performed with a Kruskal–Wallis one-way ANOVA on ranks. No statistical differences (ns) compared to worms without compound treatment (-). **(D)**. Developmental rate of wild-type worms grown in the absence (-) or presence of 10 and 50 μM of SI (left) and MSI (right). A color code was used to represent each of the following animal stages: L1–L3: early larval stages, L4: last larval stage, > L4: adult stage. Animal classification was evaluated at the indicated time points (24, 48, and 72 h). Data are represented in a stacked bar chart as mean ± SEM. No statistical differences (ns) were found compared to worms without compound treatment (-) (One-way ANOVA for MSI and One-way ANOVA on ranks for SI, *n* = 4 independent experiments).

To improve the likelihood of success in drug development, it is vital to focus the initial screening on compounds with potentially appropriate pharmacokinetic characteristics (Hay et al., 2014). For this reason, *in silico* prediction of bioavailability and ADME (absorption, distribution, metabolism, and excretion) parameters have become a common practice during the early stages of pharmaceutical development (Dahlin et al., 2015). Hence, first, we used the SwissADME online tool [Swiss Institute of Bioinformatics (SIB)] (Daina et al., 2017; Bakchi et al., 2022) to estimate the bioavailability of five water-soluble imidazole derivatives. The software analysis showed that all the derivatives have a bioavailability score greater than zero, which means that their pharmacokinetic properties are comparable to most drugs with proven effectiveness in humans (Table 1) (Martin et al., 2005; Khan et al., 2018; Masood and Fatima, 2018; Bojarska et al., 2020). In addition, except for compound number 5, all derivatives show high gastrointestinal absorption (Table 1). We also estimated the oral bioavailability radar for these compounds. This measurement qualitatively assesses six physicochemical properties that are relevant for oral bioavailability (lipophilicity, size, polarity, solubility, flexibility, and saturation) and indicates the probability that a compound could be an oral drug candidate. At first glance, we observed that the bioavailability radar for most compounds falls entirely within the region accepted for oral drug physicochemical properties (pink zone, Figure 1A). The high polarity of compound number five slightly distorts its oral bioavailability radar. Nevertheless, even for this compound the estimated area mostly overlaps with the expected one. Since these *in silico* estimates allow us to glimpse adequate overall bioavailabilities for each of the compounds, we decided to evaluate their pharmacological action. Specifically, we analyzed their effect on the OS resistance of wild-type worms exposed to the oxidizing agent FeSO_4_. We found that exposure of wild-type worms to 10 μM of compound 1 (1-(3-Sulfonatopropyl) imidazolium (SI)) and compound 3 (1-Mesityl-3-(3-sulfonatopropyl) imidazolium (MSI)) significantly increased their resistance to OS (Figure 1B). In contrast, no statistically significant changes were detected in animals exposed to the other compounds, even at higher concentrations (100 μM) (Figure 1B; Supplementary Figure S1).

**TABLE 1:** Drug-likeness parameters of the analyzed compounds. Predictions were obtained after introducing SMILES structures in the SwissADME web server. Bioavailability score, Gastrointestinal (GI) adsorption and the Lipinski’s, Ghose’s, Veber’s, Egan’s and Muegge’s rules define the drug-likeness score for each compound.

Compound	Bioavailability score	GI adsorption	Rules				
			Lipinski	Ghose	Veber	Egan	Muegge
1	0.56	High	Yes	Yes	Yes	Yes	No; 1 violation: MW < 220
2	0.56	High	Yes; 0 violation	Yes	Yes	Yes	Yes
3	0.56	High	Yes; 0 violation	Yes	Yes	Yes	Yes
4	0.56	High	Yes; 0 violation	Yes	Yes	Yes	Yes
5	0.11	Low	Yes; 1 violation: MW > 500	No; 4 violations	No; 1 violation	No; 2 violations	No; 2 violations

### 3.2 SI and MSI Protective Mechanisms Involve Specific Signaling Molecules

It has been shown that compounds with significant Redox activities could directly react and reduce oxidative molecules, therefore enhancing the resistance to OS (Devagi et al., 2018). Thus, we performed *in vitro* measurements of the ORP for these derivatives. The values obtained were 67.0 mV for **1**, 70.2 mV for **2**, 44 mV for **3**, 34,7 mV for **4** and 83.5 mV for **5**. These positive values indicate that the reduction potential of these compounds is extremely limited suggesting that the enhanced OS resistance induced by compounds **1** and **3** (hereafter referred to as SI and MSI, respectively) is not likely due to direct antioxidant action.

An alternative mechanism conferring stress resistance could be reduced food intake and the consequent activation of dietary restriction programs. Indeed, *C. elegans* mutants with diminished pharyngeal function exhibit reduced food intake and increased stress resistance and lifespan (Lakowski and Hekimi, 1998). To assess whether these drugs exert their protective effect by modulating feeding behaviors, we analyzed the pharyngeal pumping rate, which is widely used as a proxy for food intake. We did not observe significant changes in pharyngeal pumping rate in the presence of either SI or MSI compared to controls (Figure 1C). Therefore, the enhanced OS resistance found in SI- and MSI-treated animals are probably not due to reduced caloric intake.

A typical trade-off between stress resistance and development rate has been reported throughout the animal kingdom (Sørensen et al., 2003; Broughton et al., 2005; Zakrzewska et al., 2011). In *C. elegans*, for example, stress-resistant strains generally have a slower development rate (Kenyon et al., 1993). We examined whether these compounds enhance stress resistance by reducing the animal growth rate. As shown in Figure 1D, no significant changes in *C. elegans* development rate were observed for both compounds.

Taking together these results suggest that the protective effects of both imidazole derivatives could implicate the modulation of specific stress response pathways rather than redox mechanisms, changes in food intake, or growth rate.

We next performed additional *in silico* studies for both derivatives. In this case, we run the SwissADME web tool to predict drug-likeness. SwissADME analyzes five different rule-based filters, with diverse ranges of properties inside of which the molecule is defined as drug-like (Daina et al., 2017). We assessed these filters for both SI and MSI. As shown in Table 1, drug-likeness is compatible with Lipinski (Lipinski, 2000), Ghose (Ghose et al., 1999), Veber (Veber et al., 2002), and Egan (Egan et al., 2000) rules. However, the Muegge rules (Muegge et al., 2001) are compatible only for MSI, possibly due to the low molecular weight of SI (Table 1). Given that MSI did not present any violation of these parameters, in this work, we focused on the molecular mechanisms underlying the stress-protective effect of this derivative.

### 3.3 MSI Regulates the HSF-1 Signaling Pathway to Increase Oxidative Stress Resistance

**FIGURE 2:**
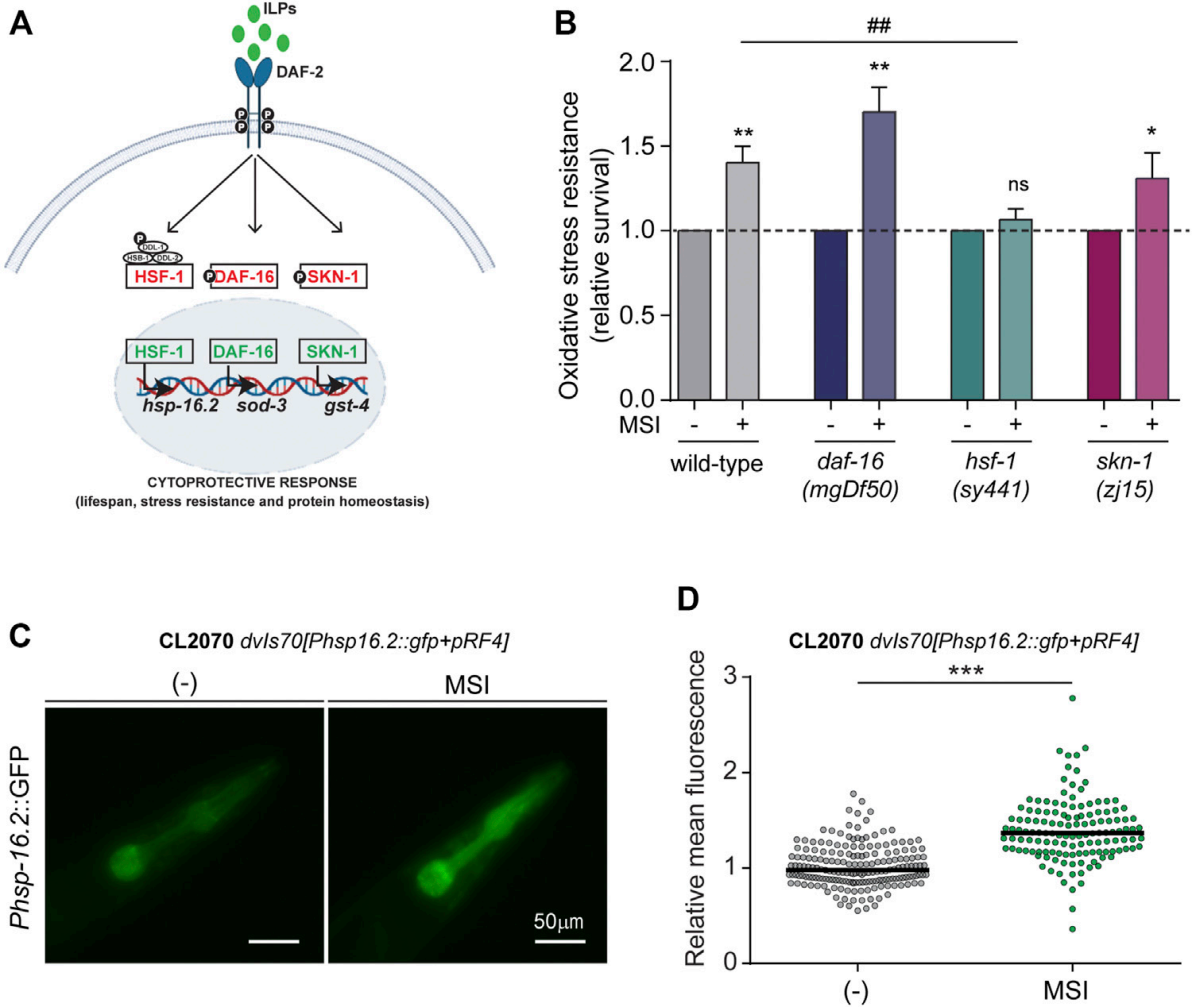
Molecular pathways involved in the mechanism of stress resistance of MSI. **(A)**. Schematic diagram showing classical transcription factors involved in the DAF-2/IIS signaling pathway. Activation of the insulin/IGF-1 receptor ortholog, DAF-2 receptor, leads to phosphorylation and cytoplasmatic sequestration of the transcription factors (TFs) DAF-16/FOXO, HSF-1/HSF, and SKN-1/Nrf2. In contrast, downregulation of the DAF-2/IIS signaling permits the nuclearization of these TFs and the triggering of cytoprotective mechanisms. **(B)**. Oxidative stress resistance (10 mM FeSO4, 1 h) of wild-type, GR1307 *daf-16(mgDf50)*, PS3551 *hsf-1(sy441)*, and QV225 *skn-1(zj15)* mutant animals exposed to MSI. Data are shown as mean ± SEM from at least 4 independent experiments (*n* = 60–80 animals per experiment per condition). Statistical differences were evaluated by comparison of means (Student’s t-test for *daf-16* animals) or medians (Mann-Whitney Rank Sum Test for wild-type and *hsf-1* worms). *represents *p*-value compared with the same strain without MSI and # represents *p*-value for wild-type and *hsf-1* mutant comparison upon MSI exposure (ns, not significant, *p < 0.05, **p < 0.01, ##*p* < 0.01). **(C)**. Representative fluorescence micrographs depicting the expression of the stress-responsive gene *hsp-16.2* in the absence and presence of 50 μM MSI. **(D)**. Quantification of *hsp-16.2* expression by measuring fluorescence intensity in the pharynx region. Data are shown in a scatter-dot plot graph, with a horizontal line indicating the median of each dataset, from five independent experiments and 25–40 animals per experiment per condition. Statistical differences were evaluated by Mann-Whitney Rank Sum Test (***p < 0.001).

We explored plausible intracellular mechanisms that could explain MSI-mediated OS protection. To this end, we focused on the cytoprotective activity of transcription factors (TFs) that act downstream of the highly conserved DAF-2/IIS such as DAF-16/FOXO, SKN-1/Nrf-2, and HSF-1/HSF1 (Murphy and Hu, 2013). These TFs activate a genetic program that ultimately confers enhanced stress resistance and longevity (Kenyon et al., 1993; Altintas et al., 2016) (Figure 2A).

We analyzed the effect of MSI on the OS resistance of mutant animals with severe deficiencies in these TFs. We first characterized resistance to iron-induced OS in *daf-16*, *hsf-1* and *skn-1* mutants in the absence of MSI. Surprisingly, while *daf-16* null mutant animals exhibit an expected decline in survival to OS, hypomorphic *hsf-1*, and *skn-1* mutants are not more prone to iron-induced stress (Supplementary Figure S2). Although downregulation of these TFs typically reduces stress resistance, several reports using RNAi silencing or hypomorphic mutants, such as the ones used here, show that resistance to some stress protocols can be unaltered or, moreover, increased (McColl et al., 2010; Baird et al., 2014; Crombie et al., 2016). While these contradictory results could be due to differences in experimental protocols, they suggest that the role of these TFs in stress resistance is much more complex than expected.

As shown in Figure 2B, exposure to 10 μM MSI enhanced OS resistance in *daf-16* and *skn-1* mutant animals, comparable to the protection observed in wild-type animals. This result suggests that MSI protective effect is independent of these TFs. Accordingly, no induction of basal levels of DAF-16 nuclear translocation (Supplementary Figure S3A) or glutathione S-transferase 4 expression (GST-4, a typical SKN-1 effector often used as a read-out for SKN-1 activity) (Supplementary Figure S3B) were observed even at higher concentrations of MSI (50 μM). It has been reported that certain conditions affecting DAF-16 nuclear translocation or *gst*-4 expression often require induction of the mechanism by a mild stressor to become apparent (De Rosa et al., 2019). However, we did not observe an increase in the expression of these TFs even under conditions that trigger these processes, such as heat (DAF-16) or juglone exposure (GST-4) (Choe et al., 2009; De Rosa et al., 2019) (Supplementary Figure S3). Moreover, in the latter case, the presence of MSI appears to decrease *gst-4* expression (Supplementary Figure S3). These results suggest that DAF-16 and SKN-1 are not implicated in the molecular mechanisms underlying the enhanced stress resistance induced by MSI.

On the other hand, MSI protective effect is abolished in *hsf-1* mutants (Figure 2B). To further reinforce these results, we assessed the expression of Heat shock protein-16.2 (HSP-16.2), an HSF-1 downstream effector widely used as a proxy for HSF-1 activation. Through microscopic analysis of transgenic worms containing an *hsp-16.2*:: Green fluorescent protein (GFP) reporter transgene, we found that MSI exposure increases fluorescence intensity in MSI-treated animals (Figures 2C,D).

Overall, these findings indicate that MSI-induced stress resistance is mediated through the HSF-1 pathway.

### 3.4 MSI Alleviates Proteotoxic-Associated Phenotypes in Transgenic Animals Expressing Aβ Peptide, α-Synuclein or Poly-Q Repeats

The impaired proteostasis and consequent accumulation of protein aggregates are common features of aging and NDs. It is also reported that both aging and NDs progression are generally associated with high levels of OS biomarkers and low levels of antioxidant defense indicators (Niedzielska et al., 2016; Cenini et al., 2019; Lévy et al., 2019; Volkert and Crowley, 2020). Since OS resistance is largely associated with a major proteostasis capacity, and MSI elicits an HSF-1-dependent cytoprotective mechanism that enhances OS resistance, we next evaluated whether this compound could reduce proteotoxicity in *C. elegans* models of protein aggregation.

**FIGURE 3:**
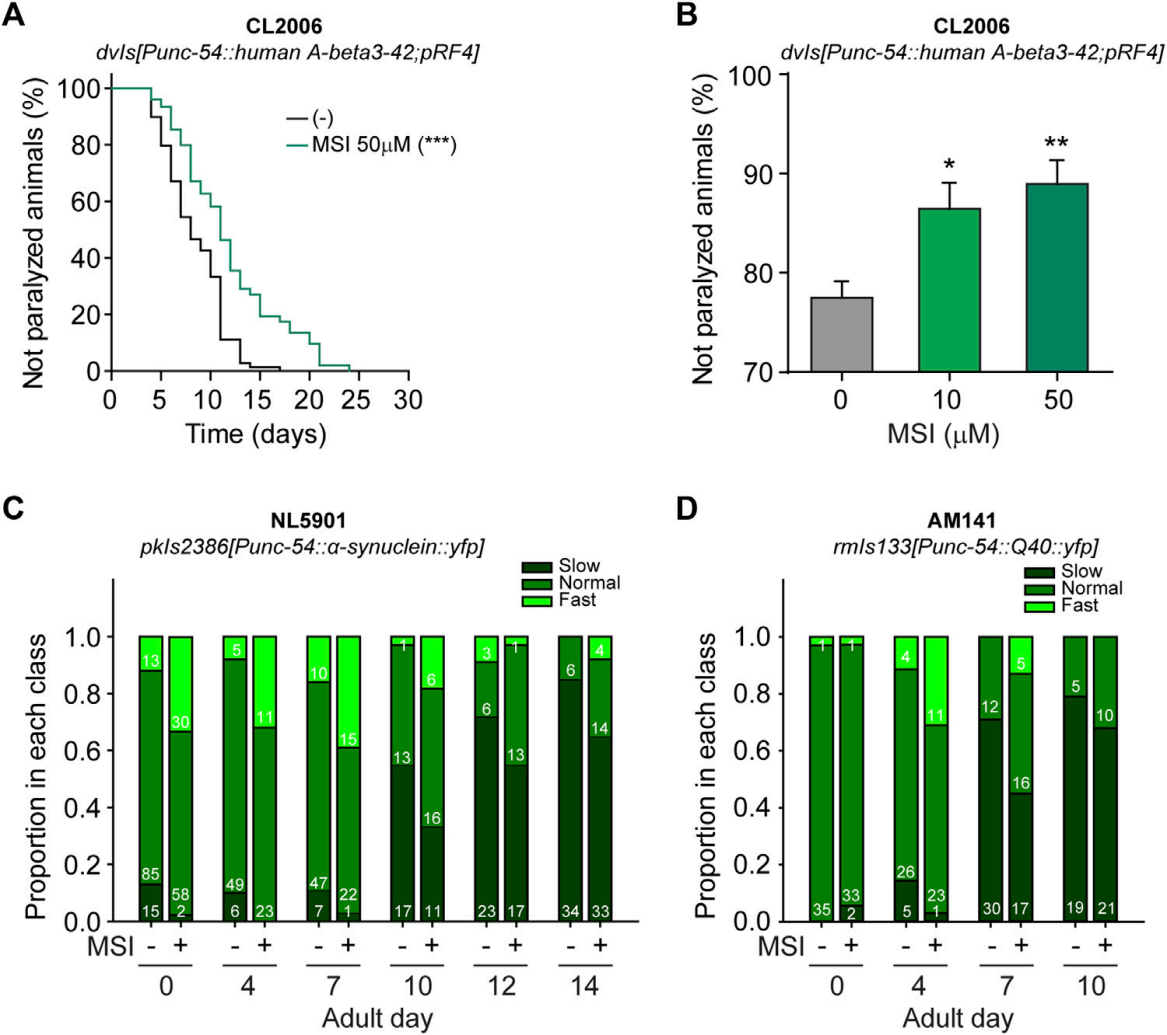
Evaluation of MSI impact on proteotoxic-associated phenotypes in *C. elegans* models of NDs. **(A)**. Influence of MSI on the paralysis of animals expressing human β-amyloid_3–42_ in muscles throughout its lifetime. Data show a representative Kaplan-Meier curve in the absence and presence of 50 μM MSI. Statistical differences were evaluated by a log-rank test (***p < 0.001). **(B)**. Evaluation of paralysis after a heat shock (35°C for 30 min). Data in the bar graph represent the mean ± SEM from 4 independent experiments with 50–80 animals per condition per experiment. Statistical significance was evaluated by One-way ANOVA followed by Holm–Sidak’s test for multiple comparisons against the control group (0 μM MSI) (*p < 0.05, **p < 0.01). **(C,D)**. Swimming behavior evaluated in *C. elegans* strains expressing α-synuclein **(C)** and poly-Q repeats **(D)** in their body wall muscles. Data were classified into three speed categories based on thrashing rates of young adult animals: normal includes those animals moving at a speed between 25% and 75% of the average thrashing rate at day 0, slow accounts for those animals moving at a speed lower than 25% of average thrashing rate of day 0 adults, and fast comprises those animals moving at a higher speed than 75% of control. Numbers inside bars show the number of animals falling in each category.

We first investigated whether MSI ameliorates locomotion phenotypes using animals that express the pathological protein involved in Alzheimer Disease (Link, 1995). The *C. elegans* homolog for the human amyloid precursor protein (APP) gene, *apl-1*, cannot produce the neurotoxic Aβ peptide (Daigle and Li, 1993). However, Aβ toxicity can be investigated by using a transgenic *C. elegans* strain expressing human Aβ under the control of a muscle-specific promoter (Link, 1995). As a result of abnormal protein aggregation in the muscle cells, this strain exhibits progressive impairment in mobility and subsequent paralysis (Link, 1995). As shown in Figure 3A, MSI-treated animals exhibit a significant delay in the typical age-dependent paralysis observed in this strain. To further confirm this result, we next performed a thermal-induced paralysis assay, as previously reported (Arya et al., 2009; Regitz et al., 2014). This assay provides an opportunity to measure positive and negative influences in Aβ-induced paralysis. As Figure 3B shows, exposure to MSI (10 and 50 μM) remarkably reduced the number of paralyzed animals upon heat shock. These results demonstrate that MSI can mitigate defects produced by the abnormal aggregation of human Aβ.

As proteotoxicity is a common hallmark of many NDs, we expanded our study to worms expressing proteins whose aggregation causes PD and HD in humans. We examined the effect of MSI in animals expressing α-syn (strain NL5901) or poly-Q (strain AM141) in body-wall muscles. Although it is not possible to directly observe neurodegeneration or specific morphological and/or behavioral neuronal defects on these animals, proteotoxicity in muscles is manifested as age-dependent locomotion defects. Therefore, these strains provide a very easily quantifiable readout to evaluate proteotoxicity *in vivo*. In addition, since muscle cells are larger than neurons, visualization of protein aggregation is also straightforward. Indeed, these strains expressing the pathological protein in muscles have been widely used to evaluate the anti-proteotoxic potential of different compounds (McColl et al., 2012; Sanchis et al., 2019; Chalorak et al., 2021).

As expected, in both strains we observed an age-dependent locomotion impairment probably due to progressive muscular damage induced by protein aggregation (Supplementary Figure S4). For instance, at adult day 4, the locomotion in liquid medium (measured as the number of thrashes/min) of NL5901 and AM141 is 67% and 77% of that of wild-type worms, respectively (Supplementary Figure S4). This locomotion impairment becomes even greater as the age of the animal increases. On day 10 of adulthood, the liquid locomotion of PD and HD animals decreased to 59% and 38% of wild-type worms of the same age (Supplementary Figure S4).

We found that exposure of NL5109 and AM141 animals to MSI reduces the proportion of animals with severely impaired motility at each of the stages tested (Figures 3C,D). Since MSI does not affect locomotion for wild-type animals, we can rule out that the drug induces hyperactivity (Supplementary Figure S5). We can therefore conclude that MSI delays the movement defects produced by the damage associated with protein aggregation of both α-syn and poly-Q repeats (see also Supplementary Figure S6).

It has been shown that clearing the pathological protein or preventing aggregate formation might be an effective strategy to ameliorate proteotoxic diseases (David et al., 2010; Bieschke, 2013; Cooper et al., 2015). Since the NL5901 and the AM141 strains express α-syn and poly-Q fused to YFP (Yellow fluorescent protein), respectively, it is possible to quantify protein aggregation by measuring mean fluorescence intensity and/or counting the number of protein aggregates (Wolozin et al., 2011).

**FIGURE 4:**
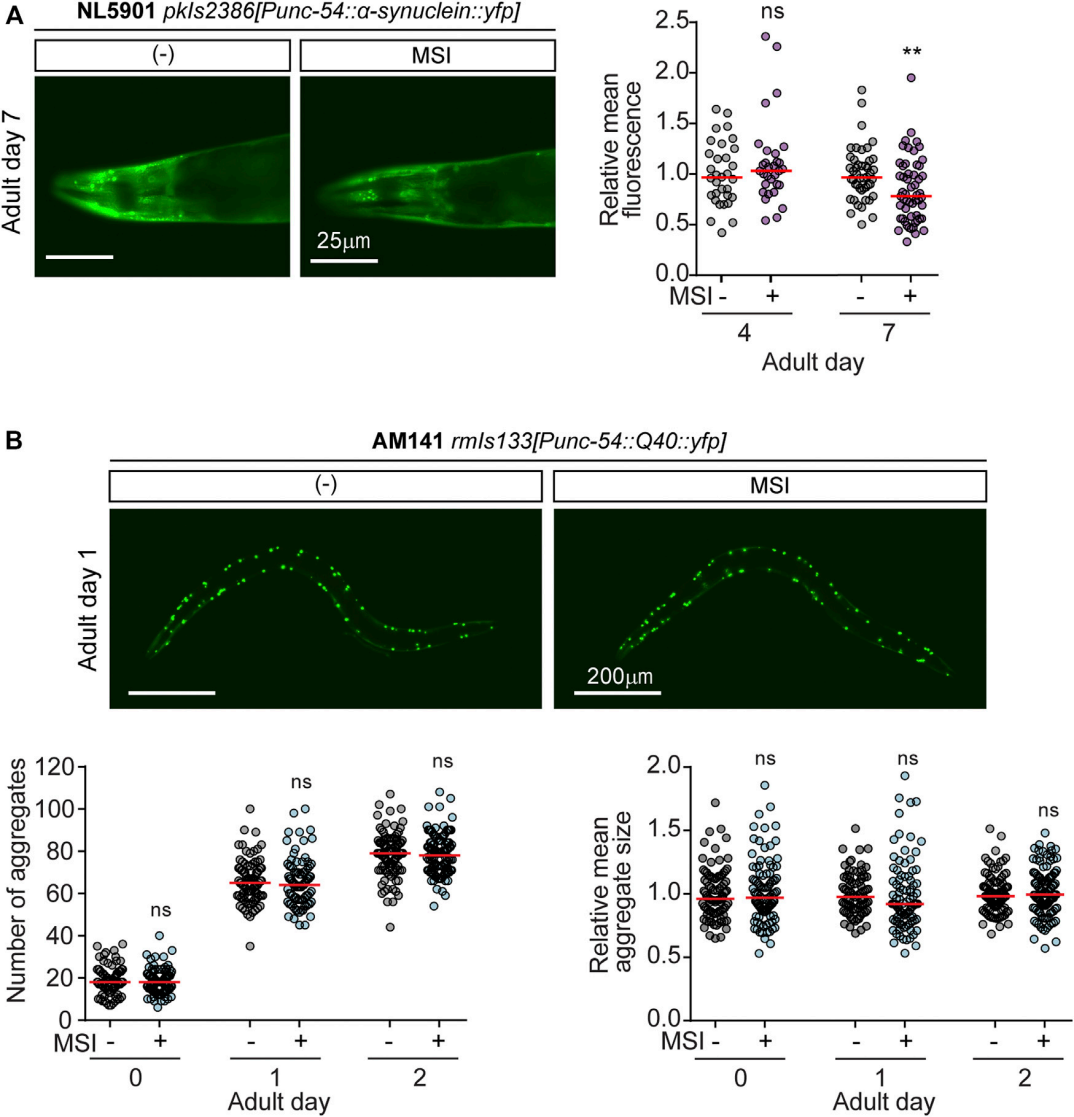
MSI effects on protein aggregation in *C. elegans* models of ND. **(A)**. Quantification of α-synuclein expression levels upon MSI treatment. Left: Representative fluorescence images depicting *α-syn::yfp* in 7-day adult worms grown in the absence or presence of 50 μM MSI. Right: Relative fluorescence intensity in the head area of the worm at different ages represented in a scatter-dot plot (line at the median). Each dot symbolizes one worm (*n* = 30–60). Statistical analysis was performed by comparing differences between median values for 0 and 50 μM MSI at each indicated stage (Mann-Whitney Rank Sum Test). Statistical symbols represent **p < 0.01 and ns not significant. **(B)** Quantification of poly-Q aggregation upon MSI treatment. Top: Representative fluorescence images depicting Q-40::YFP in 1-day adult worms grown in the absence or presence of 50 μM MSI. Bottom: The number of aggregates per animal and their relative mean size at days 0, 1, and 2 of adulthood were quantified using ImageJ FIJI and represented in their corresponding scatter-dot plot (line at the median, *n* = 60–80). Statistical analysis was performed by comparing differences between the median for 0 vs. 50 μM MSI (Mann-Whitney Rank Sum Test, ns not significant).

To analyze α-syn deposition, we quantified fluorescence intensity in the head region of NL5901 animals exposed to MSI. As shown in Figure 4, the mean fluorescence of α-syn deposits was significantly reduced in adult worms (day 7) exposed to MSI (Figure 4A). These results could implicate that the improved motility observed in MSI-treated animals may be due to an inhibition of α-syn protein oligomerization and deposition.

The AM141 strain expresses *poly-Q::yfp* in body muscles (Morley et al., 2002). In these animals, we quantified the number of aggregates at younger stages, since old animals accumulate many aggregates, and their individualization becomes troublesome. Strikingly, MSI does not seem to reduce the number or size of aggregates (Figure 4B). Thus, we can conclude that the improvement in locomotion, is not dependent on an effect on the number or size of poly-Q aggregates.

**FIGURE 5:**
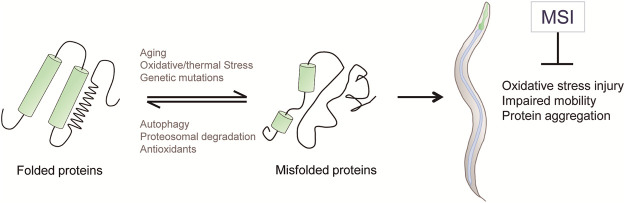
Schematic diagram depicting the cytoprotective activities of the imidazolium salt MSI, supporting its role as an antiproteotoxic agent. Cellular protein quality control systems, such as autophagy, proteosome and antioxidants, act to prevent or degrade misfolded proteins. When the capacity of these protective mechanisms is overwhelmed, abnormal proteins can accumulate into toxic protein aggregations that generate cellular damage. MSI reduces oxidative stress injury and pathological phenotypes associated with proteotoxicity accumulation.

Taken together, our results indicate that, besides enhancing OS resistance, MSI delays the onset of proteotoxicity-associated phenotypes in worms expressing proteins whose aggregation is pathognomonic of different NDs in humans (Figure 5).

## 4 Discussion

Alzheimer’s disease (AD), Parkinson’s disease (PD), and Huntington’s disease (HD) are considered among the top ten most lethal diseases (G7 Academies’ Joint Statements 2017). Despite exhaustive efforts from the scientific community, there is still no cure and no effective therapeutic strategies to mitigate their deleterious effects. Therefore, there is an urgent need for more innovative approaches to hasten the development of therapies for these disorders.

Invertebrate models, such as *C. elegans*, have emerged as a convenient strategy, not only to study the molecular basis of certain proteotoxic diseases but also to perform drug screens in a relatively short time frame.

Since the late 1960s, the pharmaceutical industry has used the imidazole nucleus to develop biologically active compounds. Recently, multi-target imidazole derivatives were postulated as candidates for the treatment of NDs (Eliewi et al., 2021). In this work, we found a compound, MSI, that delays the onset of typical locomotor defects in animals over-expressing the proteins that cause AD, HD and PD in humans.

Our results showed that MSI protects wild-type animals against oxidative stress (OS). The increase in stress resistance may be secondary to alterations in general physiological processes. One of the main conserved mechanisms underlying stress resistance and lifespan extension is dietary restriction (Fontana et al., 2010; Swindell, 2012). In *C. elegans,* feeding depends on a neuromuscular pump, the pharynx, that captures food (bacteria) and pumps it to the intestine. Alterations in pharyngeal pumping lead to reduced food intake, which inhibits the DAF-2/IIS signaling pathway, increasing stress resistance and extending animals’ lifespan (Kenyon et al., 1993). Our results demonstrate that exposure to MSI do not affect pharyngeal pumping. While this suggests that the protective effect is not mediated by reduced food intake, processes acting downstream to pharyngeal pumping may still trigger dietary restriction phenotypes (e.g., a decreased nutrient absorption in the intestine). However, diet restriction has been shown to delay growth and extend the lifespan of a broad range of animals, including *C. elegans* (Johnson et al., 1984; Vanfleteren and Braeckman, 1999; Greer and Brunet, 2009). The absence of developmental delay in animals exposed to MSI supports the notion that this compound enhances stress resistance without stimulating a typical dietary restriction state.

More than 35 human diseases are associated with protein aggregation and misfolding. Several works reported that cellular and physical stress, such as heat or oxidation, can trigger protein misfolding, unfolding, and aggregation, thus fostering the onset of proteinopathies (Morimoto, 2008; Gidalevitz et al., 2011; Morimoto, 2011; Vasconcellos et al., 2016; Kumsta et al., 2017). In fact, the saturation of endogenous antioxidant defenses was reported as one of the most important factors involved in the pathogenesis and progression of NDs (Jenner, 2003; Blesa et al., 2015; Hasanbašić et al., 2018; Nandi et al., 2019). To get insights into the mechanisms underlying MSI effect, we evaluated the role of three key transcription factors (TF) involved in stress resistance, longevity, and proteostasis from worms to mammals: SKN-1/Nrf-2, DAF-16/FOXO, and HSF-1/HSF1 (Hemdan and Almazan, 2008; Rodriguez et al., 2013; Leja-Szpak et al., 2015). Our results strongly suggest that MSI enhances stress resistance by inducing the HSF-1 pathway. On the other hand, we did not observe evidence of the activation of DAF-16 or SKN-1 upon MSI treatment. Since these 3 TFs are activated when DAF-2 signaling decreases, it is clear that this compound does not produce a general down-regulation of the insulin pathway.

Studies on *C. elegans* (Servello and Apfeld, 2020) and other organisms such as yeast *Saccharomyces cerevisiae* (Raitt et al., 2000) and the plant *Arabidopsis thaliana* (Liu et al., 2011) also highlighted the conserved importance of HSF-1 in resistance to OS. However, it is not clear how MSI induces HSF-1 activity. Since MSI can be considered a mild oxidant, it could be possible that the enhancement of OS resistance depends on an hormetic effect triggered by mild oxidation during animal exposure to the compounds. However, three of the imidazolium compounds exhibit similar ORPs values compared to that of MSI but they do not increase animal stress resistance. Moreover, it has been observed that mild OS induces the activity not only of HSF-1 but also of DAF-16 and SKN-1 (De Rosa et al., 2019). Under this scenario, a specific mechanism for HSF-1 induction is more likely to occur. In this sense, several compounds, such as Geldanamycin, have been proven to indirectly induce HSF-1 by inactivating HSP-90 (Kim et al., 1999). It would be interesting to assess if a similar mechanism underlies the MSI effects.

We also found that MSI delays the onset of locomotion phenotypes in *C. elegans* expressing α-syn, Aβ peptide or poly-Q in their muscle cells. Moreover, MSI induces the HSF-1 dependent chaperone HSP-16.2, suggesting activation of the heat shock response (HSR). It has been shown in *Drosophila* models of poly-Q diseases that HSF-1-activating compounds can reduce poly-Q-triggered toxicity through the induction of multiple molecular chaperones (Fujikake et al., 2008). Moreover, several studies in different models have also demonstrated that upregulation of chaperones, either by pharmacological induction or overexpression, decreases protein aggregation toxicity (Muchowski et al., 2000; Sittler et al., 2001; Auluck et al., 2002; Hoshino et al., 2011; Chafekar et al., 2012; Holmes et al., 2014; Nagy et al., 2016). Similarly, it is possible that MSI up-regulates HSF-1-dependent chaperones involved in the HSR, leading to decreased proteotoxicity and delayed onset of locomotion phenotypes in *C. elegans* models of protein aggregation. Supporting this idea, high levels of HSP-16.2 promote the sequestration, degradation, or refolding of abnormal oligomers, thus suppressing *in vivo* toxicity of the Aβ peptide in worms (Fonte et al., 2008). Since *C. elegans* does not naturally produce the amyloid peptide, it is possible that this chaperone could affect the multimerization/aggregation pathways of many proteins that can potentially form toxic oligomers (Fonte et al., 2008). This general function of HSR chaperones could explain the general anti-proteotoxic effect of MSI regardless of the protein causing the proteotoxicity.

Beyond chaperones induction, activation of HSF-1 can increase stress resistance by stimulation of non-classical effectors such as Aspartyl protease-4 (ASP-4)/Cathepsin and Death-associated protein kinase-1 (DAPK-1)/DAPK (Servello and Apfeld, 2020). ASP-4 is an aspartyl protease related to cathepsins D and E that have been implicated in AD (Syntichaki et al., 2002). Likewise, RNAi knockdown of *asp-4 in C. elegans* enhanced the paralysis rate and caused higher Aβ accumulation (Florez-McClure et al., 2007). Therefore, MSI effects could also involve pathways not related to classical chaperone induction.

As expected, PD animals treated with MSI show a significant decrease in α-syn aggregation. Strikingly, in animals expressing poly-Q repeats in their muscles, neither the number of aggregates nor their size is significantly altered by MSI treatment, suggesting that locomotion improvement is not dependent on poly-Q aggregate number/size. This paradox can be due to methodological limitations since many aggregation intermediates cannot be resolved (Cooper et al., 2018). However, it has been shown that the structure and solubility of the aggregates could be more critical for proteotoxicity than their number or size (Xi et al., 2016; Chiti and Dobson, 2017; De et al., 2019). For example, some oligomers could expose hydrophobic residues and unpaired structures making them highly interactive with other proteins, with critical components of the proteasome, and even with membranes (Kayed et al., 2003; Olzscha et al., 2011; Winner et al., 2011; Kim et al., 2016; Woerner et al., 2016). Moreover, large fibrillar aggregate deposits do not always correlate with disease onset or severity (Kayed et al., 2003; Leitman et al., 2013; Chiti and Dobson, 2017). In many cases, large amyloid aggregates exert a protective effect by sequestering the more-toxic oligomeric species (Saudou et al., 1998; Arrasate et al., 2004; Douglas et al., 2009; Kim et al., 2016). Thus, it is possible that while aggregation still occurs in MSI-treated animals (or changes cannot be experimentally detected), these aggregates are less proteotoxic, leading to the delayed onset of locomotion defects.

In conclusion, our work demonstrated that a water-soluble imidazole derivative, MSI, protects wild-type worms against OS and reduces proteotoxicity in *C. elegans* protein aggregation models. Despite the differences in the proteins that form aggregates among ND diseases, MSI evokes a general protective mechanism that prevents the formation or helps the degradation of more harmful aggregates. This mechanism involves the activation of the HSF-1 pathway.

Taking advantage of the biological versatility of imidazole compounds and the power of *C. elegans* to model human diseases, we have identified MSI as a potential therapeutic agent that may delay proteotoxicity *in vivo*. It is now necessary to test whether this protective effect observed in *C. elegans* could be extended into vertebrate models of proteinopathies.

## Data Availability

The original contributions presented in the study are included in the article/Supplementary Material, further inquiries can be directed to the corresponding authors.

## Abbreviations

ND, Neurodegenerative diseases; AD, Alzheimer’s disease; PD, Parkinson’s disease; HD, Huntington’s disease; MSI, 1-Mesityl-3-(3-sulfonatopropyl) imidazolium; SI, 1-(3-Sulfonatopropyl) imidazolium; HSF-1, heat shock transcription factor; DAF-16/FOXO, DAF-16/ ortholog of human Forkhead box protein O; SKN-1/Nrf2, transcription factor skinhead-1/ ortholog of human NF-E2-related factor 2 Nrf2; DAF-2/IIS, DAF-2/ insulin/insulin-like growth factor 1 signaling; TFs, transcription factors; Aβ, Amyloid-beta; α-syn, Alpha-synuclein; OS, oxidative stress; GST-4, Glutathione S-transferase 4; HSP-16.2, Heat shock protein hsp-16.2; poly-Q, Polyglutamine; ORP, oxidation-reduction potential.
