# Supplementary material for: 1-Mesityl-3-(3-Sulfonatopropyl) Imidazolium Protects Against Oxidative Stress and Delays Proteotoxicity in C. elegans
Source: Front Pharmacol. 2022 May 24;13:908696. doi: 10.3389/fphar.2022.908696 (PMC9171001; doi:10.3389/fphar.2022.908696)
Supplement: Supplementary file 1 [file DataSheet3.docx]

***Supplementary Methods***

**1-Mesityl-3-(3-sulfonatopropyl) imidazolium protects against oxidative stress and delays proteotoxicity in *C. elegans***

***Natalia Andersen^1,2^, Tania Veuthey^1,2*^, María Gabriela Blanco^1,2^, Gustavo F. Silbestri^3^, Diego Rayes^1,2*^, and María José De Rosa^1,2*^***

^1^ Instituto de Investigaciones Bioquímicas de Bahía Blanca (INIBIBB) CCT UNS-CONICET. Bahía Blanca, Argentina

^2^ Dpto de Biología, Bioquímica y Farmacia, Universidad Nacional del Sur. Bahía Blanca, Argentina

^3^ INQUISUR, Departamento de Química, Universidad Nacional del Sur, UNS-CONICET. Bahía Blanca, Argentina

*** Correspondence:**María José De Rosa
[mjderosa@criba.edu.ar](mailto:mjderosa@criba.edu.ar)

Diego Rayes

drayes@criba.edu.ar

***Supplementary Methods***

**Subcellular DAF-16 localization**

DAF-16 nuclear translocation was analyzed using a strain containing the translational *Pdaf-16::daf-16a/b::gfp* reporter. L4 animals at basal conditions or previously exposed to mild stress (35ºC, 15 min), were mounted to analyze DAF-16 distribution under a fluorescence microscope.  The number of GFP-labeled nuclei per animal was quantified by using Image J FIJI software.

**GST-4 expression**

GST-4 expression was analyzed in a transgenic strain containing a transcriptional GFP reporter in wild-type background (see strain list in main text).

To study GST-4 expression, 16 h post-L4 worms (adult day 1) were analyzed at different conditions: i) basal conditions and ii) after exposing animals to the oxidant juglone (38 μM) for 1 h followed by 3h of recovery at 20°C. GFP fluorescence from whole-body was measured using ImageJ FIJI software.
