# Supplementary material for: 1-Mesityl-3-(3-Sulfonatopropyl) Imidazolium Protects Against Oxidative Stress and Delays Proteotoxicity in C. elegans
Source: Front Pharmacol. 2022 May 24;13:908696. doi: 10.3389/fphar.2022.908696 (PMC9171001; doi:10.3389/fphar.2022.908696)
Supplement: Supplementary file 2 [file DataSheet2.docx]

***Supplementary Figures***

**1-Mesityl-3-(3-sulfonatopropyl) imidazolium protects against oxidative stress and delays proteotoxicity in *C. elegans***

***Natalia Andersen^1,2^, Tania Veuthey^1,2*^, María Gabriela Blanco^1,2^, Gustavo F. Silbestri^3^, Diego Rayes^1,2*^, and María José De Rosa^1,2*^***

^1^Instituto de Investigaciones Bioquímicas de Bahía Blanca (INIBIBB) CCT UNS-CONICET. Bahía Blanca, Argentina

^2^ Dpto de Biología, Bioquímica y Farmacia, Universidad Nacional del Sur. Bahía Blanca, Argentina

^3^ INQUISUR, Departamento de Química, Universidad Nacional del Sur, UNS-CONICET. Bahía Blanca, Argentina

*** Correspondence:**María José De Rosa
[mjderosa@criba.edu.ar](mailto:mjderosa@criba.edu.ar)

Diego Rayes

drayes@criba.edu.ar


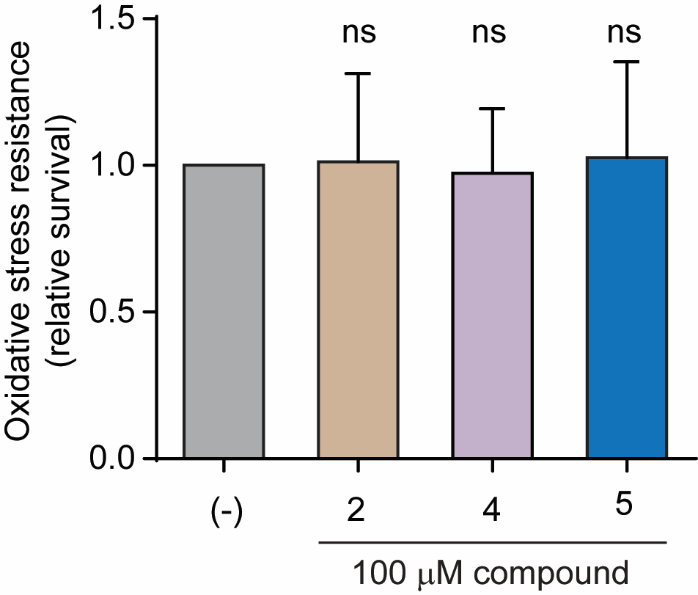


**Supplementary Figure S1.** **Effect of high concentration of compounds 2, 4 and 5 on oxidative stress resistance**

Relative survival to oxidative stress (10 mM FeSO_4_, 1h) of wild-type worms in the absence (-) or presence of 100 μM of each indicated imidazolium salt (60-80 animals per condition per experiment, n=3-4). Data in bar graph are represented as mean ± SEM. Statistical significance was evaluated by One-way ANOVA (Kruskal–Wallis test), ns, not statistically significant.


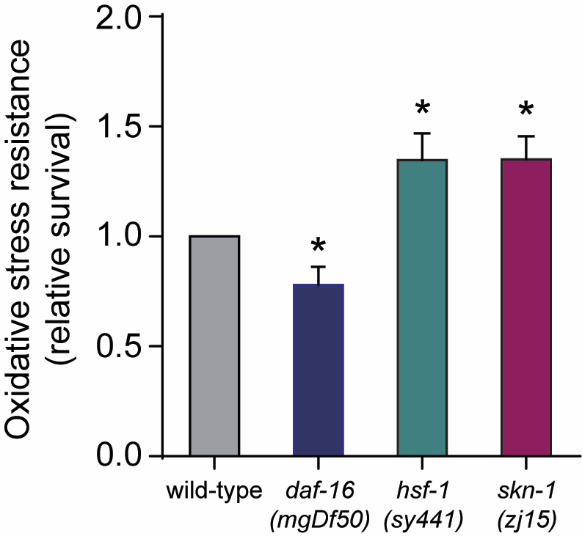


**Supplementary Figure S2.**

Oxidative stress resistance of wild-type, *daf-16(mgDf50)*, *hsf-1(sy441)* and *skn-1(zj15)* mutant animals (60-80 animals per condition per experiment, n=8-15). Oxidative stress was induced by 10mM FeSO4 for 1h. Survival was scored immediately after this treatment. Data are normalized to the resistance of wild-type animals assayed the same day. Data in the bar graph are represented as mean ± SEM. Statistical significance was evaluated by One-way ANOVA followed by Kruskal-Wallis One-Way ANOVA on ranks (**p*<0.05).

**
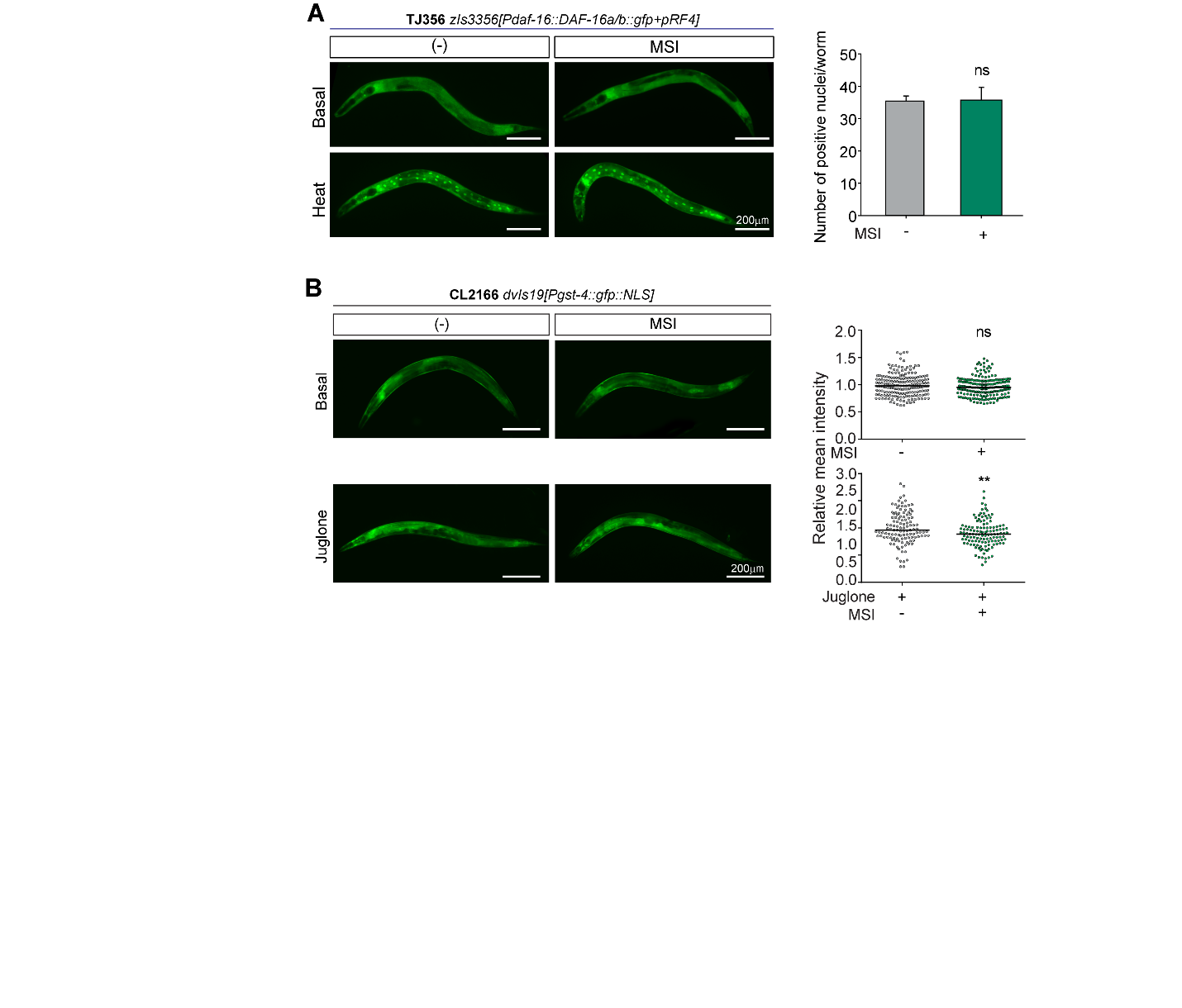
**

**Supplementary Figure S3. Molecular pathways involved in the mechanism of stress resistance of MSI**

**A.** Representative fluorescence images depicting the localization of DAF-16::GFP under basal conditions (top) and upon a short exposure to heat (35 °C, 15 min) in the absence or presence of 50 μM MSI (left) (strain TJ356 *zIs3356[Pdaf-16::DAF-16a/b::gfp+pRF4]*). Quantification of the number of cells with nuclear DAF-16 per animal is depicted in the graph bar (right) (n=12-15 animals per condition). Statistical differences were evaluated by Mann-Whitney Rank Sum Test (ns, not significant). **B**. Representative fluorescence micrographs of transgenic worms expressing GST-4::GFP (strain CL2166 *dvIs19[Pgst-4::gfp::NLS]*. Different conditions were analyzed: basal (top) and juglone (38 μM 1 h, bottom panel). Quantification of *gst-4::gfp* is shown as scatter-dot plot graphs, with a horizontal line at the median (data from 3-5 independent experiments with 30-40 animals per condition per experiment). Statistical differences were evaluated by Mann-Whitney Rank Sum Test (ns, not significant; ***p*<0.01).


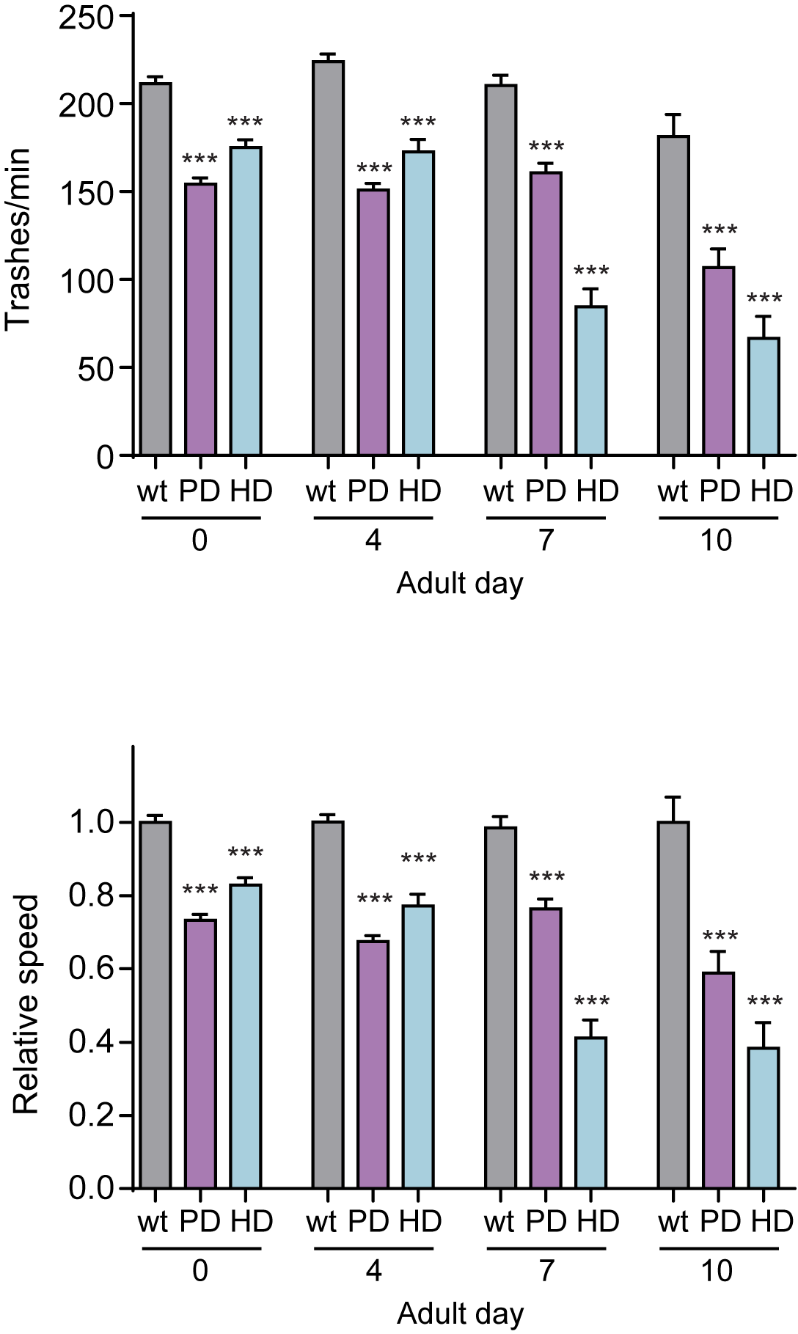


**Supplementary Figure S4. Validation of *C. elegans* models of proteotoxicity and neurodegeneration**

Swimming behavior in increasing animal ages. Bar graph depicting relative speed calculated as the percentage of thrashes/min of wild-type worms from the same age. wt, PD and HD are abbreviations of wild-type, PD strain (NL5901) and HD strain (AM141), respectively. Grey, purple and light blue is the color code for depicting wt, PD, and HD swimming rate. Statistical differences were evaluated by Kruskal-Wallis One-Way ANOVA on ranks (****p*<0.001).


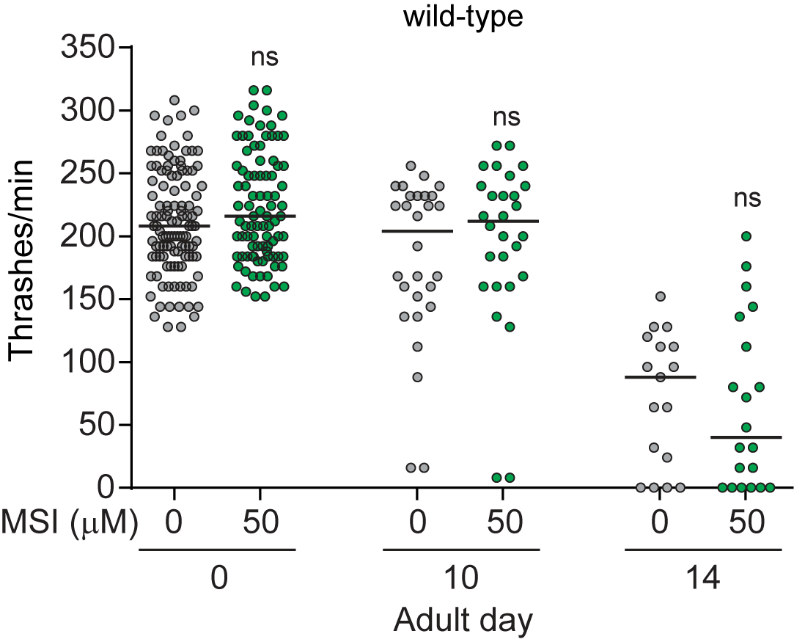


**Supplementary Figure S5. Swimming behavior of wild-type animals in the presence of MSI**

Swimming behavior is not affected by 50 μM of MSI in either young or aged wild-type animals. Data are shown as scatter-dot plots at the indicated age (line at the median). Each dot symbolizes one worm, with >16 animals per condition. Statistical analysis was performed by comparing differences between median values for 0 and 50 μM MSI at each indicated stage (Mann-Whitney Rank Sum Test), ns not significant.

**
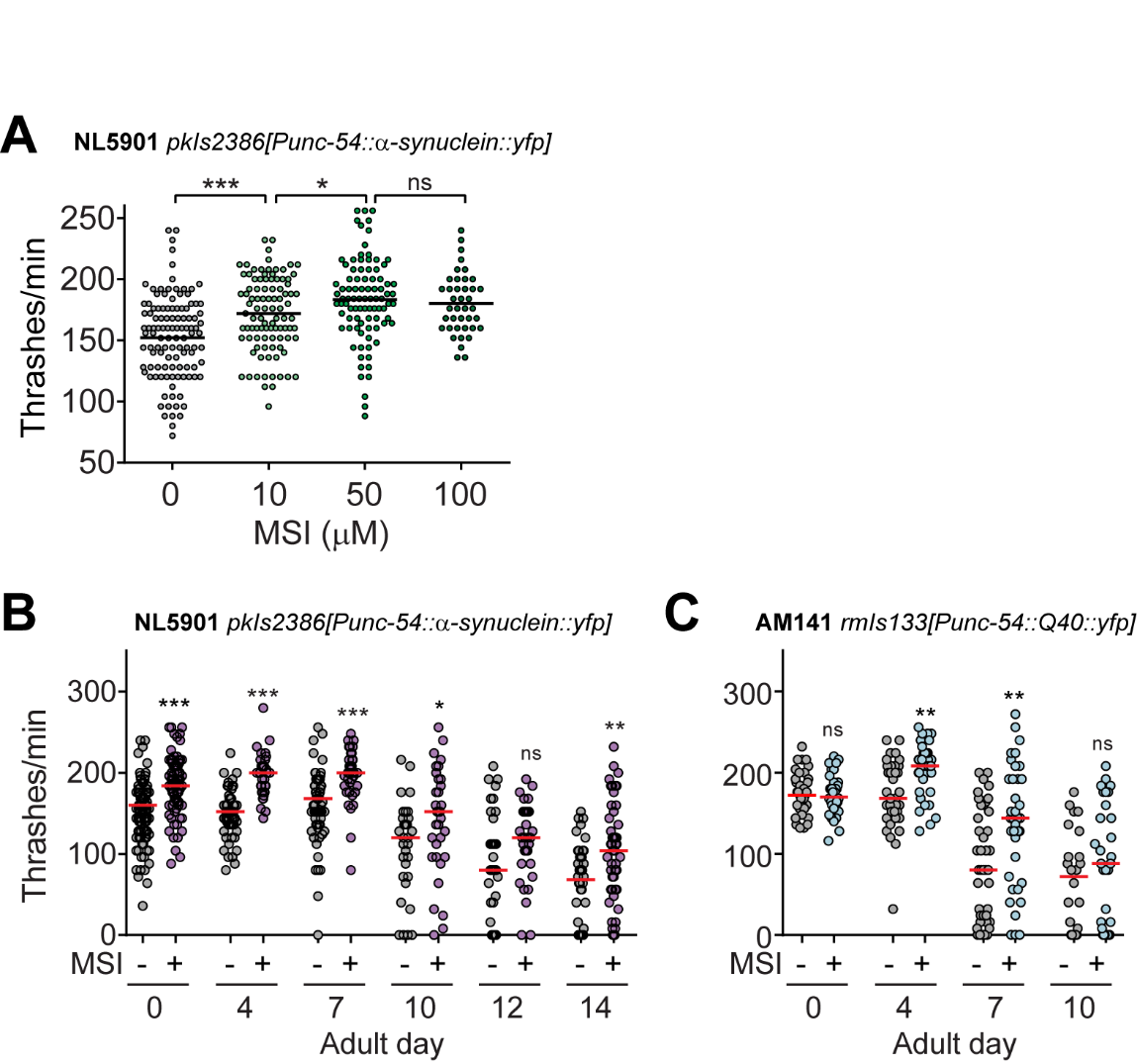
**

**Supplementary Figure S6. Effect of MSI in the swimming behavior of *C. elegans* models of α-synuclein and poly-Q protein aggregation**

**A.** Swimming behavior of the *C. elegans* model of α-synuclein proteotoxicity at increasing MSI concentrations. L4 animals from the model strain NL5901 *Punc-54::α -synuclein::yfp* were used. Data are shown as scatter-dot plots at the indicated MSI concentrations (line at the mean). Each dot symbolizes one worm, with > 40 animals per condition. Statistical analysis was performed by comparing differences between the mean number of thrashes per minute between every condition and the subsequent higher MSI concentration (Student’s t-test). Statistical symbols represent **p*<0.05, ****p*<0.001 and ns not significant. **B-C.** Animals from NL5901 (B) and AM141 (C) strains were grown under the presence of 0 or 50 μM MSI. Data are shown as scatter-dot plots at the indicated ages (line at the median). Each dot symbolizes one worm, with > 30 animals per condition. Statistical analysis was performed by comparing differences between 0 and 50 μM MSI at each indicated stage. Student’s t-test was used for comparing NL5901 animals on adult days 4, 10 and 12 and AM141 worms on adult day 0. Mann-Whitney Rank Sum Test was employed for the remaining comparisons. Statistical symbols represent **p*<0.05, ***p*<0.0,1, ****p*<0.001 and ns not significant.
