# Supplementary material for: 1-Mesityl-3-(3-Sulfonatopropyl) Imidazolium Protects Against Oxidative Stress and Delays Proteotoxicity in C. elegans
Source: Front Pharmacol. 2022 May 24;13:908696. doi: 10.3389/fphar.2022.908696 (PMC9171001; doi:10.3389/fphar.2022.908696)
Supplement: Supplementary file 3 [file DataSheet1.docx]

***Supplementary Data***

**1-Mesityl-3-(3-sulfonatopropyl) imidazolium protects against oxidative stress and delays proteotoxicity in *C. elegans***

***Natalia Andersen^1,2^, Tania Veuthey^1,2*^, María Gabriela Blanco^1,2^, Gustavo F. Silbestri^3^, Diego Rayes^1,2*^, and María José De Rosa^1,2*^***

^1^Instituto de Investigaciones Bioquímicas de Bahía Blanca (INIBIBB) CCT UNS-CONICET. Bahía Blanca, Argentina

^2^ Dpto de Biología, Bioquímica y Farmacia, Universidad Nacional del Sur. Bahía Blanca, Argentina

^3^ INQUISUR, Departamento de Química, Universidad Nacional del Sur, UNS-CONICET. Bahía Blanca, Argentina

*** Correspondence:**María José De Rosa
[mjderosa@criba.edu.ar](mailto:mjderosa@criba.edu.ar)

Diego Rayes

drayes@criba.edu.ar

***Supplementary Data***

**Supplementary Data**

**Table of Contents**

| Title page and detailed list of contents of the Supporting Information (SI) | 2 |
| --- | --- |
| ^1^H and ^13^C NMR of 1-(3-sulfonatopropyl)imidazolium (**1**) | 3 |
| ^1^H and ^13^C NMR of 1-Methyl-3-(3-sulfonatopropyl)imidazolium (**2**) | 5 |
| ^1^H and ^13^C NMR of 1-Mesithyl-3-(3-sulfonatopropyl)imidazolium (**3**) | 6 |
| ^1^H and ^13^C NMR of 1-(2,6-Diisopropylphenyl)-3-(3-sulfonatopropyl)imidazolium (**4**) | 7 |
| ^1^H and ^13^C NMR of 1,3-bis(2,6-diisopropyl-4sodiumsulfonatophenyl)imidazolium (**5**) | 8 |

1-(3-sulfonatopropyl)imidazolium (**1**) (^1^H-NMR, D_2_O)

1-(3-sulfonatopropyl)imidazolium (**1**) (^13^C-NMR, D_2_O)

1-(3-sulfonatopropyl)imidazolium (**1**) (^1^H-NMR, DMSO-d_6_)

**
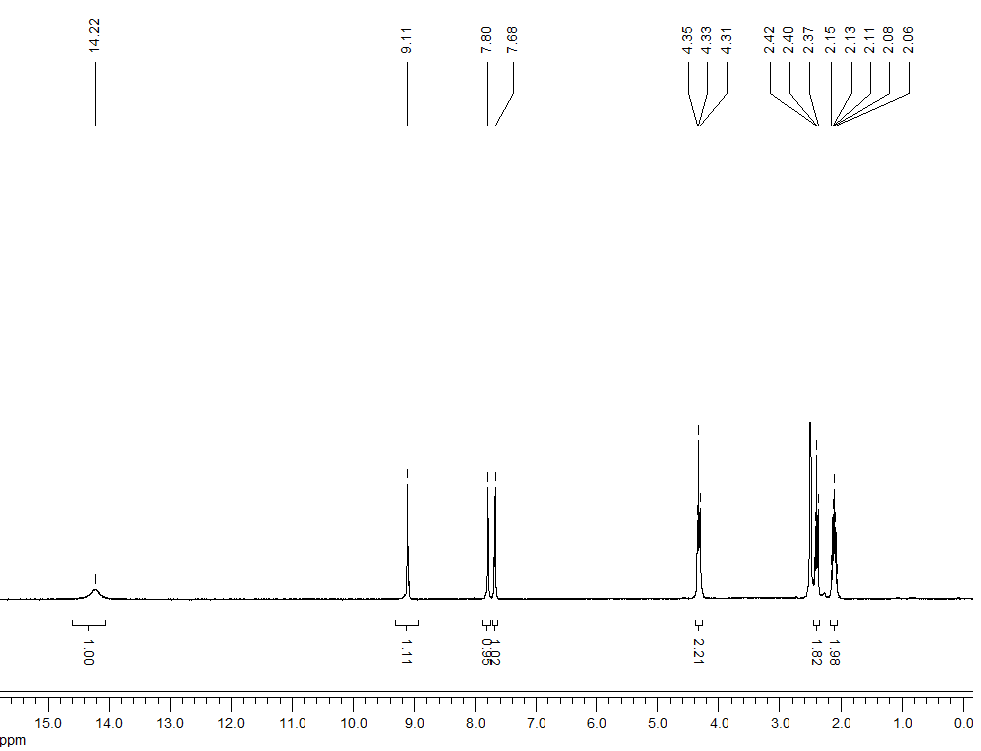
**

1-(3-sulfonatopropyl)imidazolium (**1**) (^13^C-NMR, DMSO-d_6_)


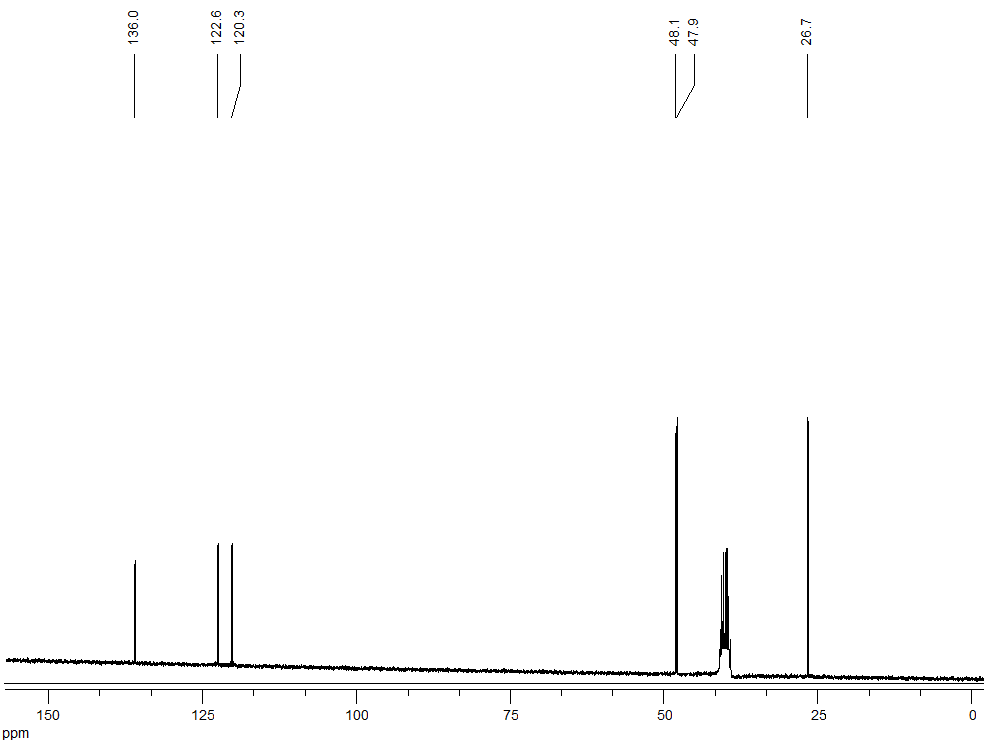


1-Methyl-3-(3-sulfonatopropyl)imidazolium (**2**) (^1^H-NMR, D_2_O)

**
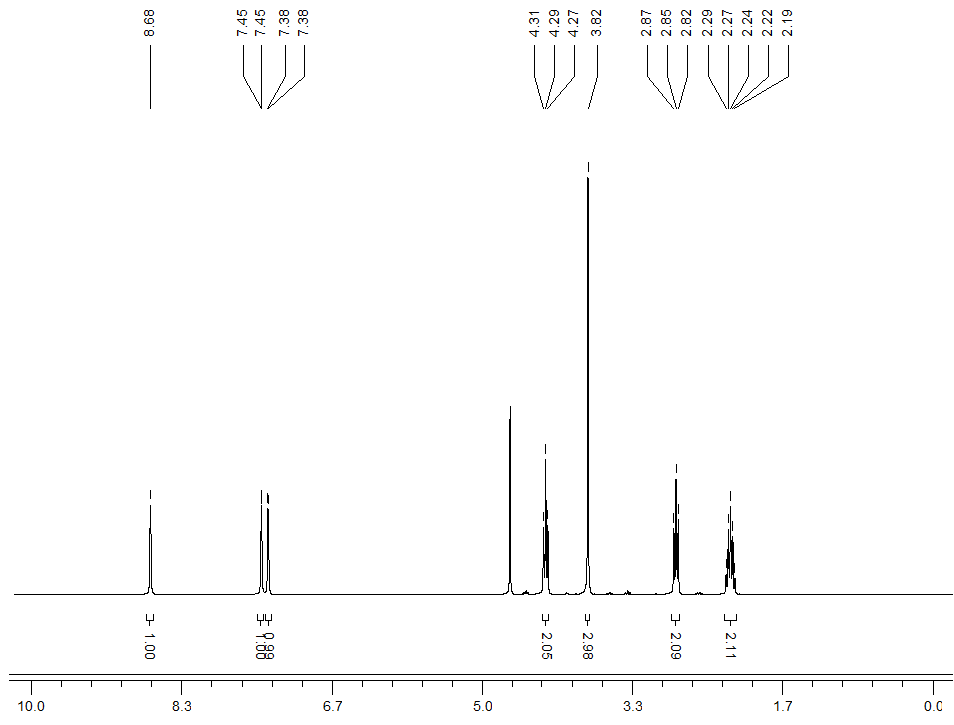
**

1-Methyl-3-(3-sulfonatopropyl)imidazolium (**2**)

**
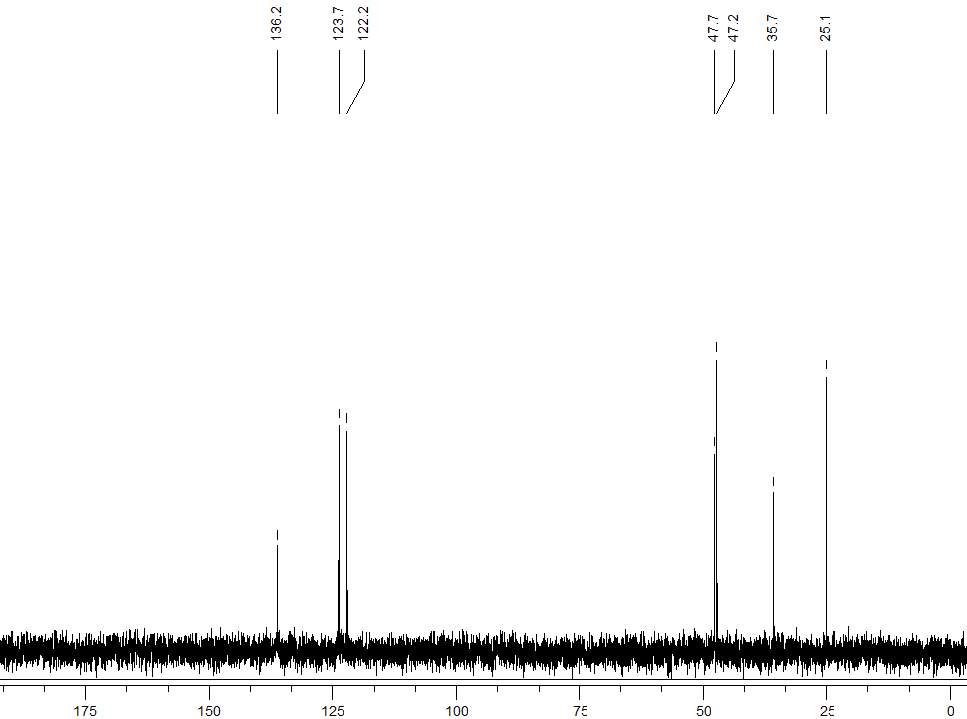
**

1-Mesithyl-3-(3-sulfonatopropyl)imidazolium (**3**) (^1^H-NMR, DMSO-D_6_)

**
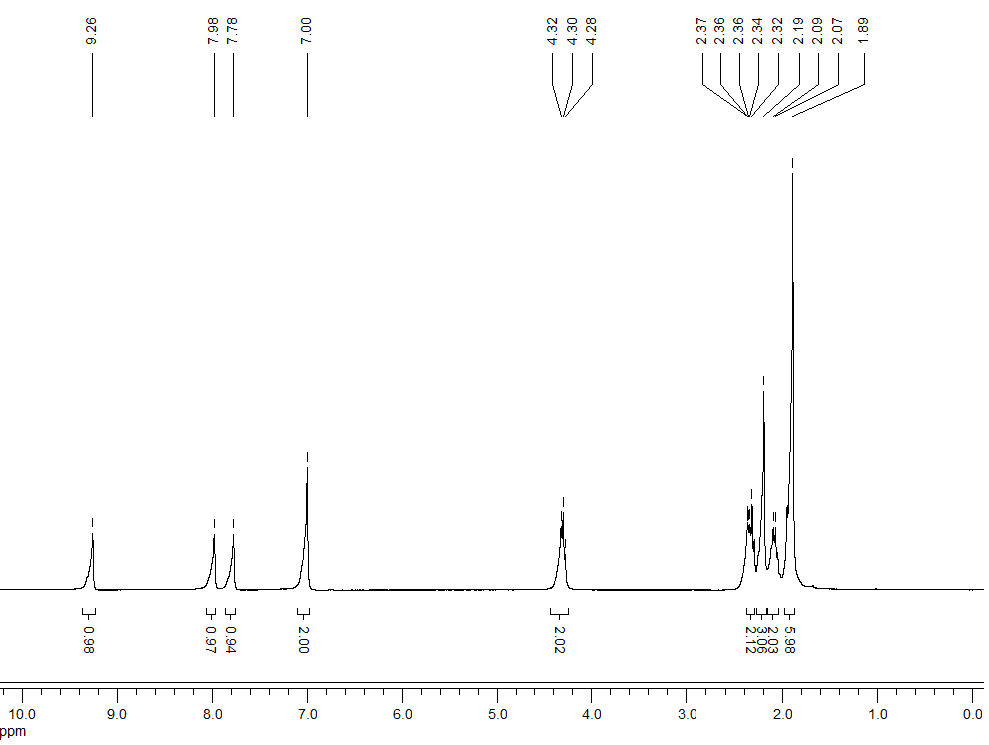
**

1-Mesithyl-3-(3-sulfonatopropyl)imidazolium (**3**) (^13^C-NMR, DMSO-d_6_)

**
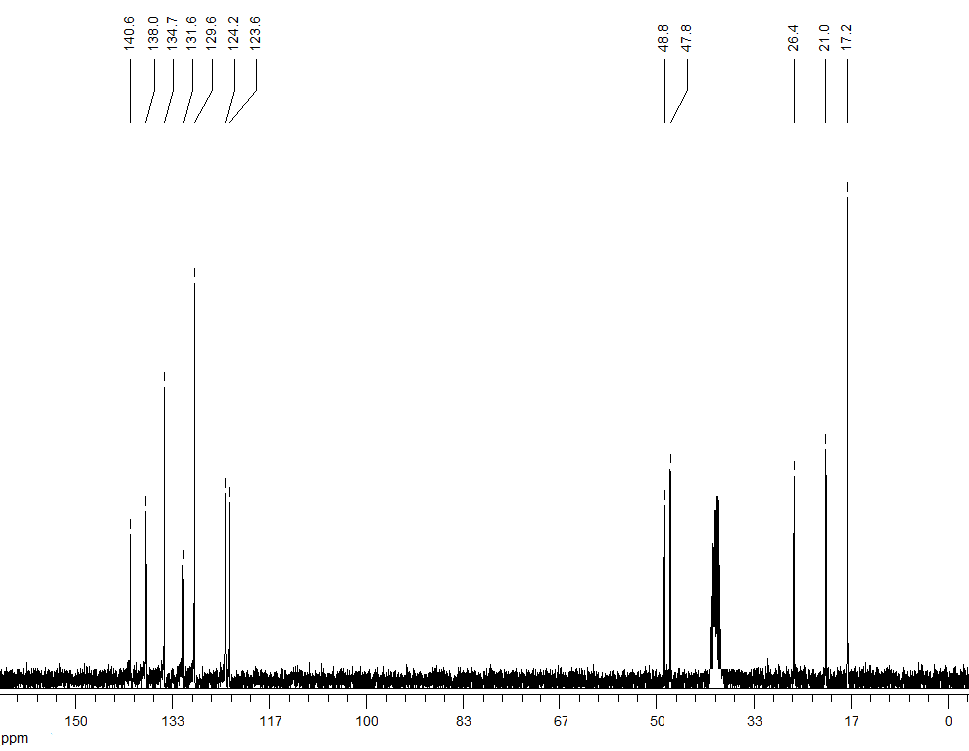
**

1-(2,6-Diisopropylphenyl)-3-(3-sulfonatopropyl)imidazolium (**4**) (^1^H-NMR, D_2_O)

**
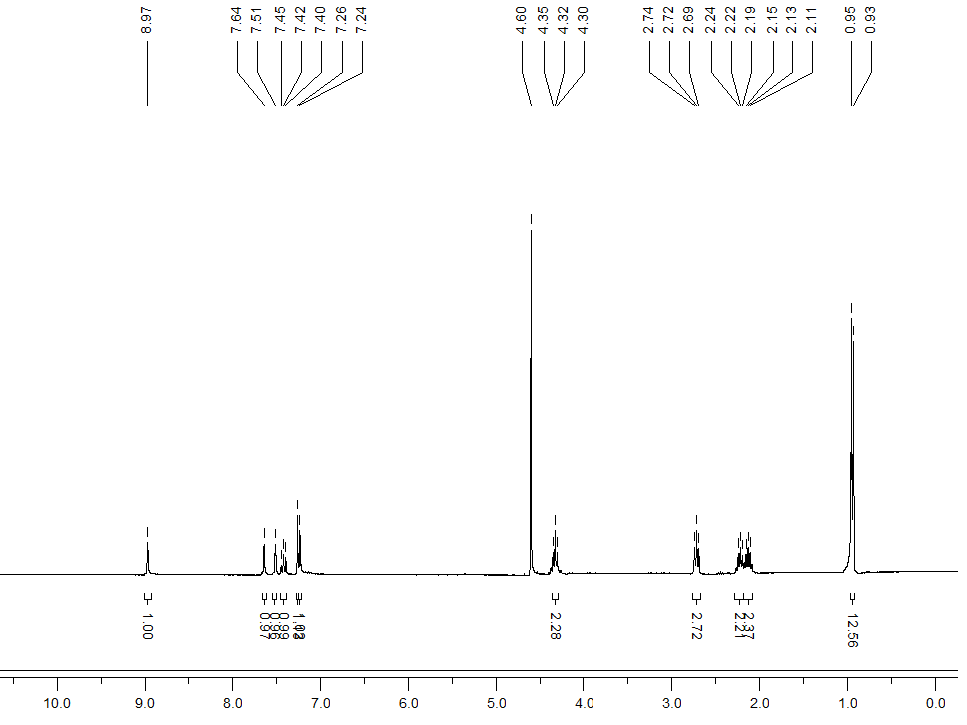
**

1-(2,6-Diisopropylphenyl)-3-(3-sulfonatopropyl)imidazolium (**4**) (^13^C-NMR, D_2_O)

**
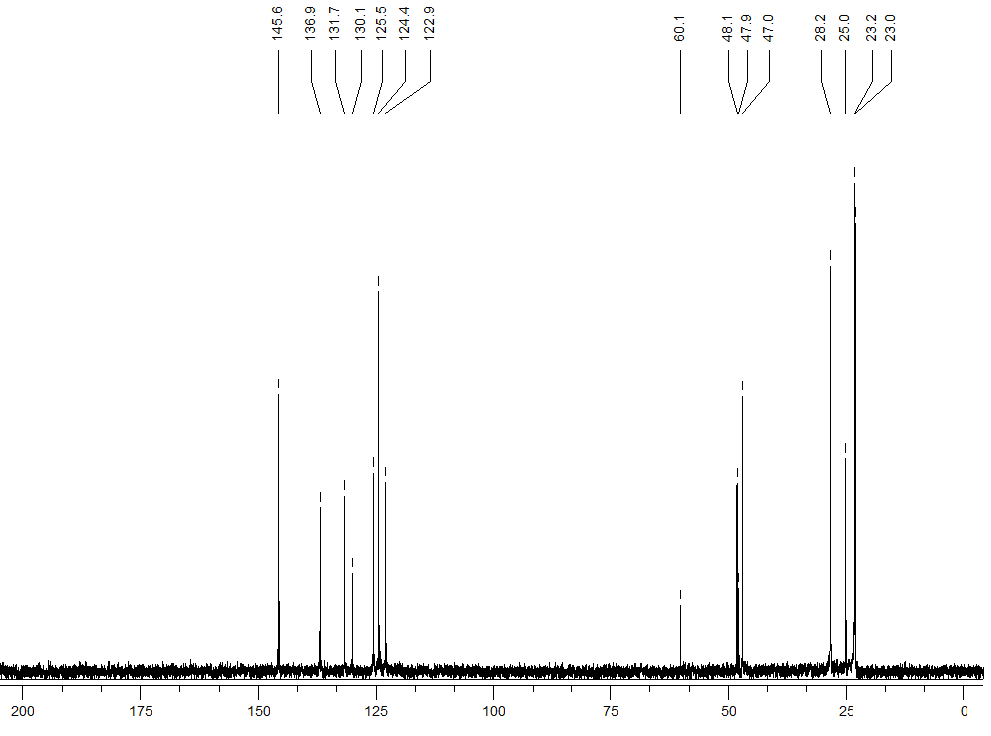
**

1,3-bis(2,6-diisopropyl-4sodiumsulfonatophenyl)imidazolium (**5**) (^1^H-NMR, D_2_O)

**
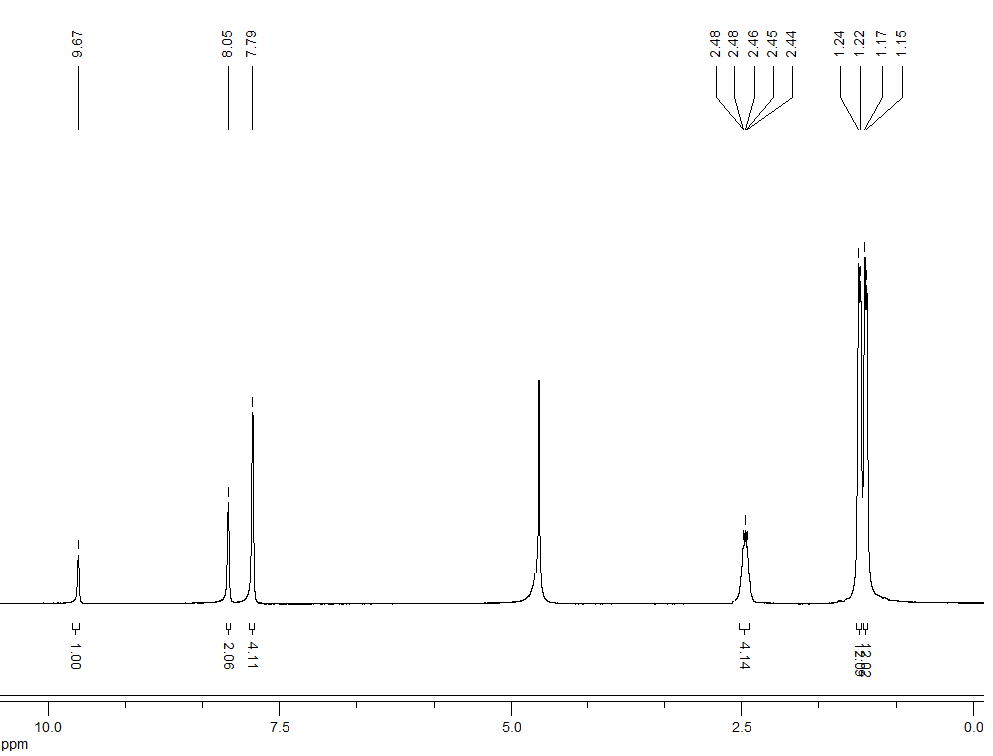
**

1,3-bis(2,6-diisopropyl-4sodiumsulfonatophenyl)imidazolium (**5**) (^13^C-NMR, D_2_O)**
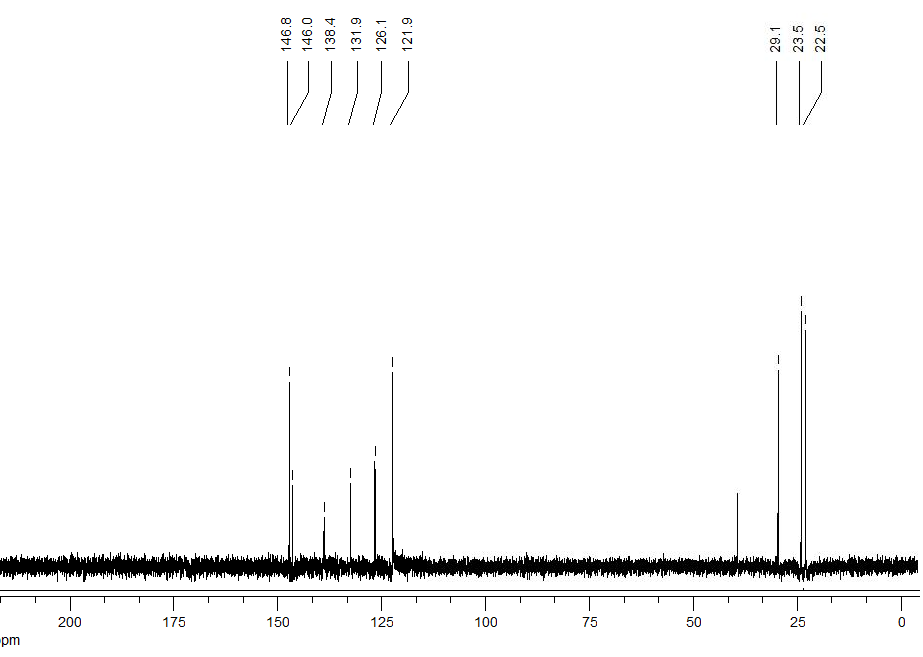
**
